# Tolerance to dietary linalool primarily involves co-expression of cytochrome P450s and cuticular proteins in *Pagiophloeus tsushimanus* (Coleoptera: Curculionidae) larvae using SMRT sequencing and RNA-seq

**DOI:** 10.1186/s12864-023-09117-7

**Published:** 2023-01-19

**Authors:** Shouyin Li, Hui Li, Cong Chen, Dejun Hao

**Affiliations:** 1 Co-Innovation Center for Sustainable Forestry in Southern China, Nanjing Forestry University, Nanjing, Jiangsu China; 2 College of Forestry, Nanjing Forestry University, Nanjing, Jiangsu China

**Keywords:** Specialist pest, Camphor trees, Terpenoid-derived metabolites, Host adaptation, Single-molecule real-time sequencing, Co-expression networks analysis

## Abstract

**Background:**

*Pagiophloeus tsushimanus* (Coleoptera: Curculionidae), an emerging forest pest exclusively infesting camphor trees, has recently caused severe ecological and economic damage in localized areas in China. Its population outbreak depends largely on the capacity to overcome the pressure of terpenoid-derived metabolites (e.g. linalool) from camphor trees. At present, the molecular basis of physiological adaptation of *P. tsushimanus* to dietary linalool is poorly understood, and there is no available reference genome or transcriptome.

**Results:**

Herein, we constructed the transcriptome profiling of *P. tsushimanus* larvae reared on linalool-infused diets using RNA sequencing and single-molecule real-time sequencing. A total of 20,325 high-quality full-length transcripts were identified as a reference transcriptome, of which 14,492 protein-coding transcripts including 130 transcription factors (TFs), and 5561 long non-coding RNAs (lncRNAs) were detected. Also, 30 alternative splicing events and 8049 simple sequence repeats were captured. Gene ontology enrichment of differential expressed transcripts revealed that overall up-regulation of both cytochrome P450s (CYP450s) and cuticular proteins (CPs), was the primary response characteristic against dietary linalool. Other physiological effects possibly caused by linalool exposure, such as increase in Reactive Oxygen Species (ROS) and hormetic stimulation, were compensated by a handful of induced genes encoding antioxidases, heat shock proteins (HSPs), juvenile hormone (JH) epoxide hydrolases, and digestive enzymes. Additionally, based on co-expression networks analysis, a diverse array of hub lncRNAs and TFs co-expressed with CYP450s and CPs were screened as the potential gene regulators. Temporal expression of candidate transcripts determined by quantitative real-time PCR also indicated a cooperative relationship between the inductions of CYP450s and CPs upon exposure to linalool.

**Conclusions:**

Our present study provides an important transcriptome resource of *P. tsushimanus*, and lays a valuable foundation for understanding how this specialist pest copes with chemical challenges in its specific host environments.

**Supplementary Information:**

The online version contains supplementary material available at 10.1186/s12864-023-09117-7.

## Background

It is well established that plants and herbivores are in a constant battle for survival [1–4]. The former keeps adapting their chemistry to defend against the latter, which sparks the rise of extensive specialized metabolites (mainly secondary metabolites) currently found in plants. Among these, terpenoids are the largest and most diverse plant metabolites and have been widely known as the defensive vehicles in conifers protection [5–10]. But actually some broad-leaved trees, as exemplified by camphor trees *Cinnamomum camphora*, are also equipped with a suite of terpenoid-derived metabolites*.* Different parts of camphor trees are abundant in monoterpenoid oxygenated derivatives such as D-camphor and linalool [11]. The evidence strongly suggests that these specialized metabolites have toxic and/or repellent effects on pests and diseases with the potential as antimicrobial or insecticidal agents [12, 13]. In general, terpenoids as important chemical mediators in plant-herbivore interactions work to the benefit of the plants and to the detriment of herbivores.

In the face of a diverse range of phytochemicals, specialization could lead to a competitive advantage by developing more fine-tuned metabolic features for digestion and detoxification of host plants [14, 15]. In this context, classic examples are that specialist herbivores feeding on a single or a few plants with terpenoid-based defenses have evolved adaptation strategies, as mainly demonstrated for pine-feeding bark beetles [16, 17]. Some bark beetles can use cytochrome P450 monooxygenases (CYP450s) to detoxify or even biotransform pine-derived terpenoids into pheromones by both sexes [18]. Progress in the molecular functions of CYP450 genes has deciphered the roles of genes belonging to the CYP6 family in response to phytochemicals including several terpenoids [19]. It also must be cautioned that exposure to terpenoids appears to disrupt the insects’ cuticular development because of their strong volatility and penetrability in analogy to mechanisms of contact insecticides or fumigants [20]. In contrast, the resistant strains of target insects have evolved to deploy a class of structural components, cuticular proteins (CPs), in order to enable the cuticle thickening that minimizes penetration of xenobiotics (e.g. contact insecticides or fumigants) into their bodies [20]. Penetration resistance referring to reduced insecticide penetration through the cuticle has been reported in a variety of insect species [21–23]. However, whether some insect pests of camphor trees have co-evolved similar or distinct mechanisms to counter monoterpenoid oxygenated derivatives (e.g. D-camphor and linalool) remains elusive so far.

More recently, an emerging insect pest, *Pagiophloeus tsushimanus* (Coleoptera: Curculionidae) has been found to persist exclusively on camphor trees, and locally cause serious damage in China [24, 25]. It evokes our great interest that how this insect pest tolerates host-specific terpenoid defenses from camphor trees without incurring apparent fitness costs. In our previous study, we tested the sensitivity of *P. tsushimanus* eggs and larvae to camphor oil and several monoterpenoids including D-camphor and linalool, suggesting that this weevil possesses various levels of tolerance to different monoterpenoids [26]. Subsequently, more work place emphasis on the mechanism underlying the tolerance of this weevil to D-camphor, and the role of this compound as a key semiochemical in host recognition (unpublished data). But also, linalool is undeniably an important source of chemical pressure when the larvae fed on the phloem of camphor trees. Published work indicated a broad spectrum of toxicity or repellent activity of linalool to some stored-product pests [13, 26–28], while little is known about how this specialist weevil overcomes the detrimental effects of linalool exposure.

Next-generation sequencing has attained considerable momentum so far, and RNA sequencing (RNA-seq) is an indispensable tool for exploring the mRNA expression profiles of target genes, and further predicting their physiological functions [29]. Studies investigating the transcriptomic responses of insect pests to insecticides and plant metabolites (including a small fraction of terpenoids) exposures have been well-established [30–34]. Single-Molecule Real-Time (SMRT) sequencing as an emerging technology overcomes the limitation of short-read sequences, and provides a full-length reference transcriptome for RNA-seq analysis of non-model insects without reference genomes [35]. Also, SMRT sequencing contributes to accurately analyzing the long non-coding RNA (lncRNA) and the structural information of sequences, such as alternative splicing (AS) and simple sequence repeat (SSR) [35]. The combination of RNA-seq and SMRT sequencing has been used in various studies to obtain more complete transcriptome information reflecting the physiological state of insects under stress environments [36–38].

Therefore, in this study we employed an integrated RNA-seq and SMRT sequencing approach to a general view of the response of *P. tsushimanus* larvae to a long-term linalool exposure (from 1st- to 3th-instar) in the dietary. AS and SSR prediction, expression analysis of lncRNA, transcription factor, and functional genes (mRNA) were then performed to determine the mechanism driving the tolerance to dietary linalool in *P. tsushimanus* larvae. Understanding of how *P. tsushimanus* adapts to a linalool-rich dietary will provide a valuable clue for the physiological mechanisms of its dietary specialization and outbreak. Our findings will aid in screening potential molecular targets for this pest management from the perspective of plant chemical defenses.

## Results

### Summary of the transcriptome data output from single-molecule real-time and Illumina sequencing

To obtain a comprehensive transcriptome database of *Pagiophloeus tsushimanus*, one library with 1–6 K cDNA insert size was generated from the pooled RNA samples (three male adults, three female adults, sixty eggs, three newly molted 1st- to 5th-instar larvae, and three pupae) using the Pacific Sequel platform. As shown in Table S1, a total of 18.31 Gb nucleotides with 16,263,362 subreads were obtained after the initial filtering process. These subreads were then subjected to circular consensus sequence (CCS) analysis, and 113,436 CCSs with a full-length non-chimera (FLNC) reads of 68,407 were produced. 26,937 consensus isoforms were screened out after the Iterative clustering of error correction (ICE). Finally, a total of 20,325 high-quality full-length transcripts with a mean length of 1194.31 bp were established as a reference transcriptome to correct the reads produced by Illumina sequencing. In addition, detailed information on the length distribution of these reads were shown in Fig. S1. For Illumina sequencing, six RNA samples (CK: A1, A2, A3; Linalool_LC15: B1, B2, B3) were separately sequenced on the Illumina HiSeq 2000 platform. We obtain 50,560,795 raw reads and 50,265,983 clean reads per sample with an average of Q20 of 98.60%, Q30 of 95.56% and GC content of 43.20%, which indicated a high accuracy and quality of the sequenced data for further analysis (Table S2).

### Structural analysis of transcripts

For alternative splicing (AS) analysis, only 30 AS events were detected without determining their types, since so far no reference genome is available for transcriptome assembly in *P. tsushimanus*. For simple sequence repeat (SSR) analysis, total of 8049 SSRs were identified from 5728 SSR-containing sequences among the 15,148 tested transcripts. Specifically, all SSR repeat types were divided into six SSR motif units, including Mono-nucleotide, Di-nucleotide, Tri-nucleotide, Tetra-nucleotide, and Penta-nucleotide motifs. Among these, the highest number of SSR motif units is Mono-nucleotide motif (7201, 89.46%), as shown in Table S3, followed by Tri-nucleotide motif (551, 6.85%) and Di-nucleotide motif (265, 3.29%).

### Functional annotation and enrichment of differentially expressed transcripts

In total, 20,325 full-length transcripts were performed into functional annotation analysis, of which 14,492 transcripts (71.30%) were annotated in at least one database (Table S4). Homologous species information in the annotation of Nr database showed that 56.6% of transcripts had the highest scoring blast matches to *Dendroctonus ponderosae*, followed by *Sitopjilus oryzae* (22.5%) (Fig. S2a). Based on GO and KEGG annotation, a total of 8623 and 12,305 transcripts were respectively classified into three main GO categories (i.e. biological processes, cellular components, and molecular functions) and six KEGG categories (i.e. human diseases, metabolism, organismal systems, cellular processes, environmental information processing, and genetic information processing), which are detailed in Fig. S2b & c.

**Fig. 1:**
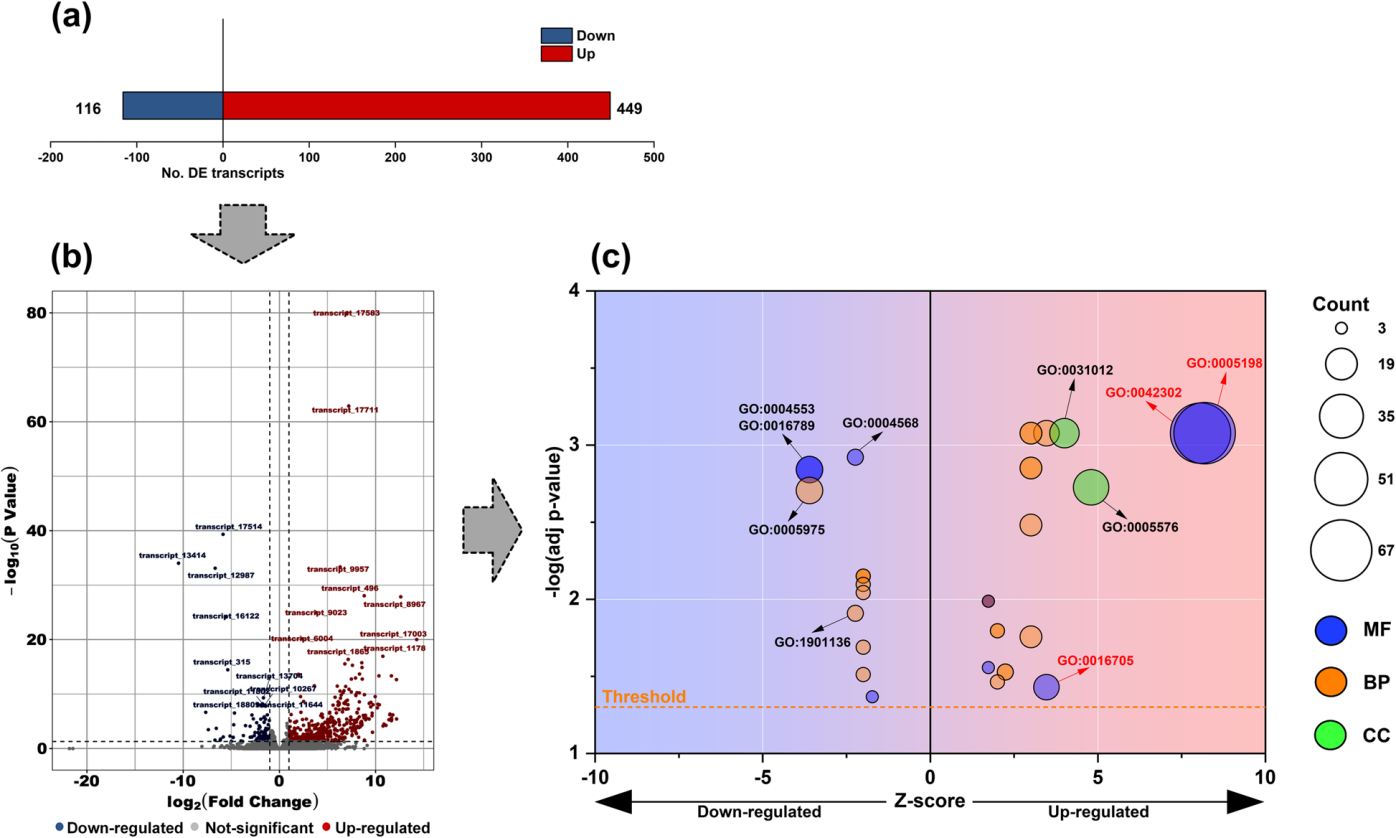
Identification and enrichment analysis of differentially expressed transcripts. (**a**) Number of significantly down-regulated and up-regulated transcripts in Linalool_LC15 group when compared with those in CK group. (**b**) Volcano plot of differentially expressed transcripts. Dotted horizontal and vertical lines indicate *P*-value thresholds and ± 2-fold change, respectively. Red and blue dots indicate significantly up-regulated and down-regulated transcripts, respectively. Names of top 10 up-regulated and down-regulated transcripts are shown. (**c**) Bubble chart of significantly functionally enriched Gene Ontology (GO) terms in up-regulated and down-regulated transcripts. Dotted horizontal line indicates *P*-value thresholds. X-axis represents Z-score [(No. up-regulated transcripts - No. down-regulated transcripts) / SQRT (No. up-regulated transcripts + No. down-regulated transcripts)]. GO terms highlighted in red are further discussed in subsequent analysis

**Fig. 2:**
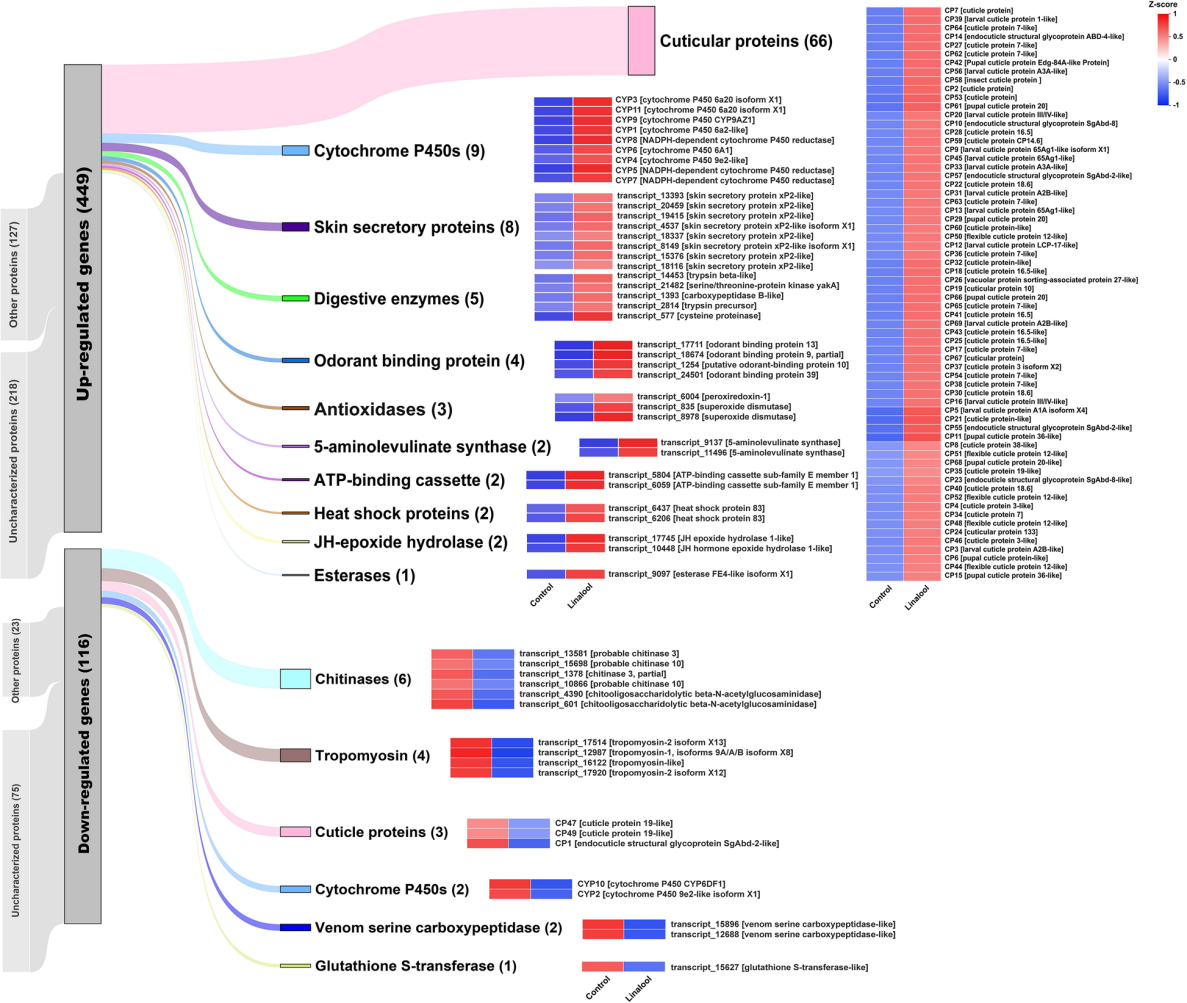
Heatmap analysis of differentially expressed transcripts of interest. Data are shown as the average values of expression for each group, and are Z-score normalized across each row. Red bars indicate up-regulated transcripts while blue bars indicate down-regulated transcripts. Detailed information on the transcript levels is listed in Table S5

To explore the functional genes involved in response to dietary linalool, we evaluated the differences in transcription levels between the linalool-exposed and control groups. The results showed that 565 differentially expressed (DE) transcripts were observed in the larvae reared on the linalool-contained diets, including 449 up-regulated transcripts and 116 down-regulated transcripts (Fig. 1a & b). Furthermore, 10 top-most up-regulated transcripts predominantly consisted of transcripts encoding cytochrome P450s (CYP450s) and cuticular proteins (CPs), while tropomyosin-associated transcripts were mainly suppressed in the exposed larvae (Table 1). GO enrichment analysis consistently revealed that the pathways related to oxidoreductase activity (GO: 0016705) and cuticular structural constituents (GO: 0005198 and GO: 0042302) were significantly activated by linalool exposure (Table 2, Fig. 1c). A total of 11 CYP450 and 69 CP transcripts that were differentially expressed in the linalool-exposed group were further identified, and most of them exhibited an up-regulation expression in varying degrees (Fig. 2). Furthermore, other inducible transcripts of interest, such as skin secretory proteins, digestive enzymes, odorant binding proteins, antioxidases, 5-aminolevulinate synthases, ATP-binding cassette, heat shock proteins, juvenile hormone (JH) epoxide hydrolases, and esterase, were targeted as functional mRNAs engaging in responses to dietary linalool (Fig. 2). Detailed information on their expression levels was provided in Table S5.

**Table 1:** Summary of the top 10 differentially expressed transcripts in linalool-exposed compared to control groups

Model	Top 10 DE transcripts	NR annotation description	Fold change (Linalool_LC15/Control)	***P***-value
Up-regulation	transcript_17583	cytochrome P450 6a20 isoform X1	132.024	9.2E-81
	transcript_17711	odorant binding protein 13	148.267	1.3E-63
	transcript_9957	cytochrome P450 6a20 isoform X1	79.760	4.5E-34
	transcript_496	cuticle protein 7-like	452.547	8.9E-29
	transcript_8967	elongation factor 2	6286.820	1.4E-28
	transcript_9023	cytochrome P450 9e2-like	14.217	1.1E-25
	transcript_17003	cuticle protein 7-like	19,771.776	9.6E-21
	transcript_6004	peroxiredoxin-1	5.139	7.1E-21
	transcript_1865	cuticle protein 7-like	142.446	4.2E-17
	transcript_1178	[uncharacterized protein]	1731.377	1.1E-17
Down-regulation	transcript_17514	tropomyosin isoform X15	0.017	4.6E-40
	transcript_13414	AgSP-1 arylphorin	0.001	9.3E-35
	transcript_12987	tropomyosin-1	0.010	7.8E-34
	transcript_16122	tropomyosin-like	0.020	7.3E-25
	transcript_315	[uncharacterized protein]	0.024	3.5E-15
	transcript_13704	nesprin-2	0.318	4.9E-10
	transcript_10267	[uncharacterized protein]	0.344	1.1E-08
	transcript_11802	nesprin-2	0.297	6.1E-09
	transcript_18809	attacin-B-like	0.282	1.5E-08
	transcript_11644	[uncharacterized protein]	0.364	1.5E-08

**Table 2:** Summary of the top 5 enriched Gene Ontology terms in differentially expressed transcripts through linalool-exposed treatment

Model	Category	Top 5 GO terms	DE transcripts count	Adj ***P-***value	Z-score
Up-regulation	MF	structural constituent of cuticle [GO:0042302]	65	0.0008	8.062
	MF	structural molecule activity [GO:0005198]	67	0.0008	8.185
	BP	extracellular region [GO:0006952]	23	0.0018	4.796
	CC	extracellular matrix [GO:0031012]	16	0.0008	4.000
	MF	oxidoreductase activity [GO:0016705]	12	0.0371	3.464
Down-regulation	MF	hydrolase activity, hydrolyzing O-glycosyl compounds [GO:0004553]	13	0.0014	−3.606
	MF	hydrolase activity, acting on glycosyl bonds [GO:0016798]	13	0.0014	−3.606
	BP	carbohydrate metabolic process [GO:0005975]	13	0.0020	−3.606
	BP	carbohydrate derivative catabolic process [GO:1901136]	5	0.0123	−2.236
	MF	chitinase activity [GO:0004568]	5	0.0012	−2.236

### Co-expression networks between lncRNA and target mRNA, and TF and target mRNA

**Fig. 3:**
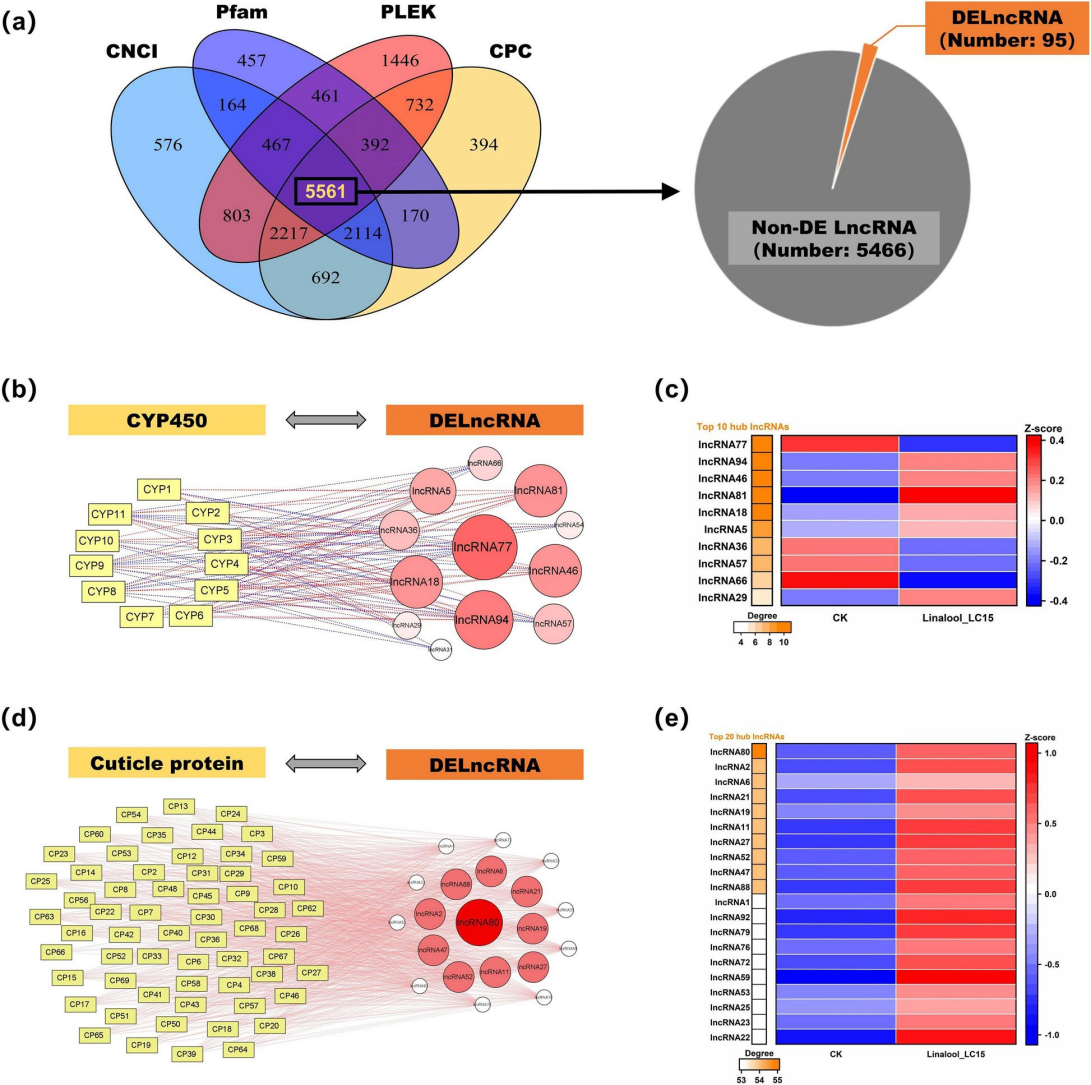
Identification of differentially expressed lncRNAs involved in regulation of cytochrome P450 (CYP450) and cuticular protein (CP) genes. (**a**) Venn diagram of showing the number of lncRNAs predicted by four methods, Coding Potential Calculator (CPC, score < 0.5), Coding-Non-Coding Index (CNCI, score < 0), PLEK (score < 0), and Pfam protein structure domain analysis (no annotation). A total of 95 differentially expressed lncRNAs (DELncRNAs) were identified for subsequent tests. (**b**, **d**) Co-expression networks between DELncRNA and CYP450 genes, DELncRNA and CP genes using Pearson correlation coefficient. Rectangle and circular nodes indicate target mRNAs (i.e. CYP450 or CP) and DELncRNAs, respectively. Red and blue lines indicate positive and negative correlations, respectively. The lncRNAs with top-ranked degrees are colored red. (**c**, **e**) Heatmap of hub lncRNAs ranked by degrees. Data are shown as the average values of expression for each group, and are Z-score normalized across each row. Red bars indicate up-regulated lncRNAs while blue bars indicate down-regulated lncRNAs

**Fig. 4:**
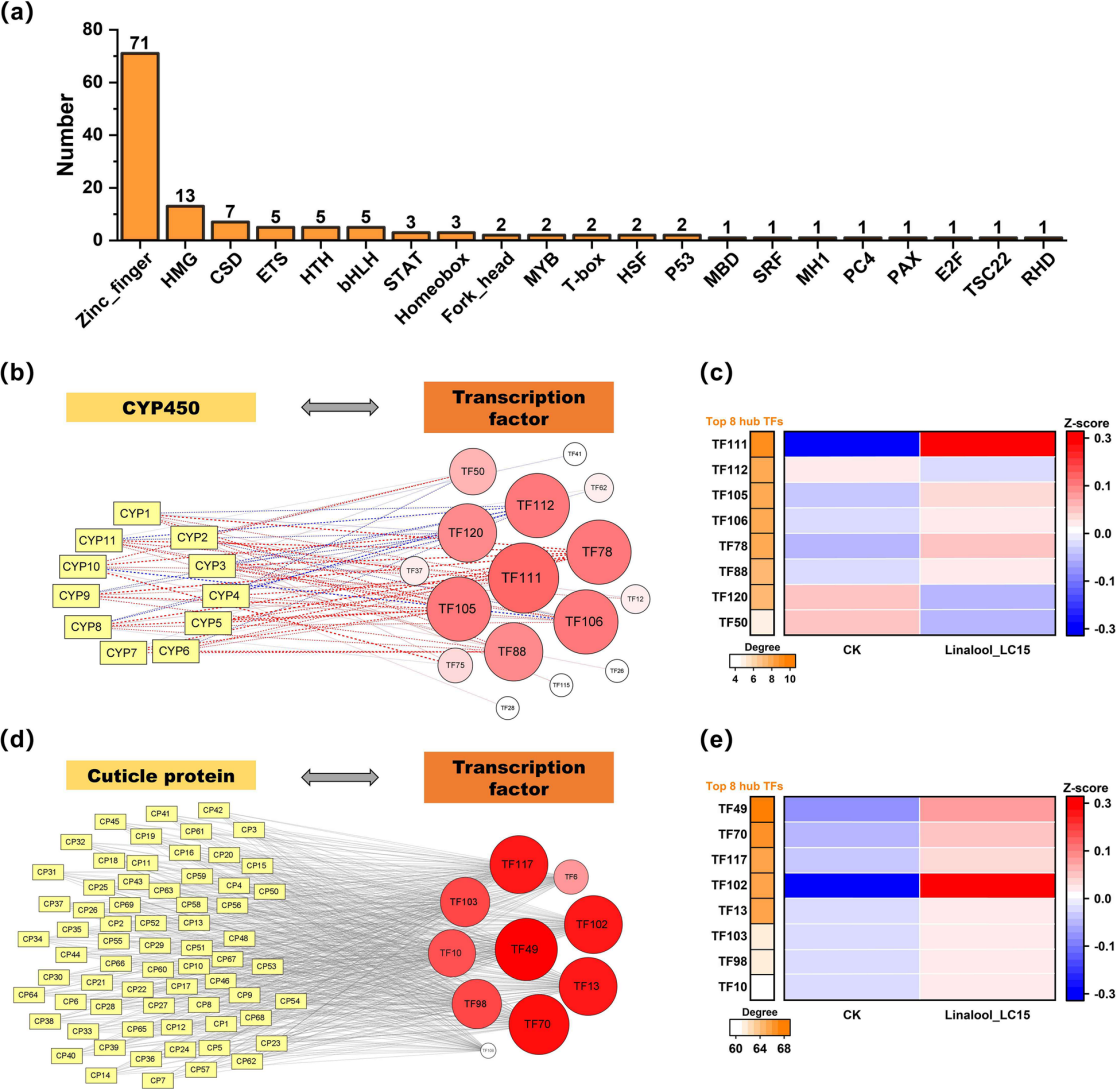
Identification of transcription factors (TFs) involved in regulation of cytochrome P450 (CYP450) and cuticular protein (CP) genes. (**a**) Statistics of TF family. (**b**, **d**) Co-expression networks between TFs and CYP450 genes, TFs and CP genes using Pearson correlation coefficient. Rectangle and circular nodes indicate target mRNAs (i.e. CYP450 or CP) and DELncRNAs, respectively. Red and blue lines indicate positive and negative correlations, respectively. The TFs with top-ranked degrees are colored red. (**c**, **e**) Heatmap of hub TFs ranked by degrees. Data are shown as the average values of expression for each group, and are Z-score normalized across each row. Red bars indicate up-regulated TFs while blue bars indicate down-regulated TFs

As an important component of full-length transcriptome, lncRNAs were identified using four coding potential analysis methods (i.e. CNCI, Pfam, PLEK, and CPC). As shown in Fig. 3a, given that intersection of four databases, a total of 5561 lncRNAs were yielded to further DE analysis, and only 95 lncRNAs were found to be differentially expressed in the larvae exposed to dietary linalool. More details of DE lncRNAs were provided in Table S6. To determine the potential relevance of biological functions between lncRNAs and target mRNAs (i.e, 11 CYP450 and 69 CP transcripts), we constructed lncRNA-mRNA co-expression networks on the basis of their expression correlations (Fig. 3b & d). In the networks, the top-ranked lncRNAs in terms of the degree values were considered as hub lncRNAs. There were inconsistent responses occurring in the top 10 hub lncRNAs related to the expression of CYP450s, where six of them were up-regulated but the other four displayed down-regulated expression (Fig. 3c). In addition, all of 20 hub lncRNAs related to the expression of CPs were markedly inducible in the treatment group (Fig. 3e). Also, 130 TF transcripts were identified using the animal TFDB version 3.0 database. Among them, 71 TF transcripts belonged to Zinc-finger family, accounting for the highest percentage (Fig. 4a). More details of TFs were provided in Table S7. Subsequently, TF-mRNA co-expression networks were established to explore the transcriptional regulatory of TFs on the target mRNAs (Fig. 4b & d). The degree values of TFs were calculated and ranked to determine the top 8 hub TFs. Moreover, the expression levels of TF111 and TF102 significantly increased in the Linalool_LC15 group, and consequently the two TFs were regarded as key regulators mediating the transcriptional expression of target mRNAs (Fig. 4c & e).

### Temporal expression of candidate lncRNAs, TFs, and mRNAs involved in response to dietary linalool

Based on samples for Illumina sequencing, we determined the relative expression levels of 16 interesting transcripts (including two lncRNAs, two TFs, six CYP450s, and six CPs) using RT-qPCR. These tested transcripts showed a positive correlation between RNA-seq and RT-qPCR data (Pearson’s *r* = 0.744, *P* < 0.001), which suggests the sequencing results are highly reliable (Fig. S3).

**Fig. 5:**
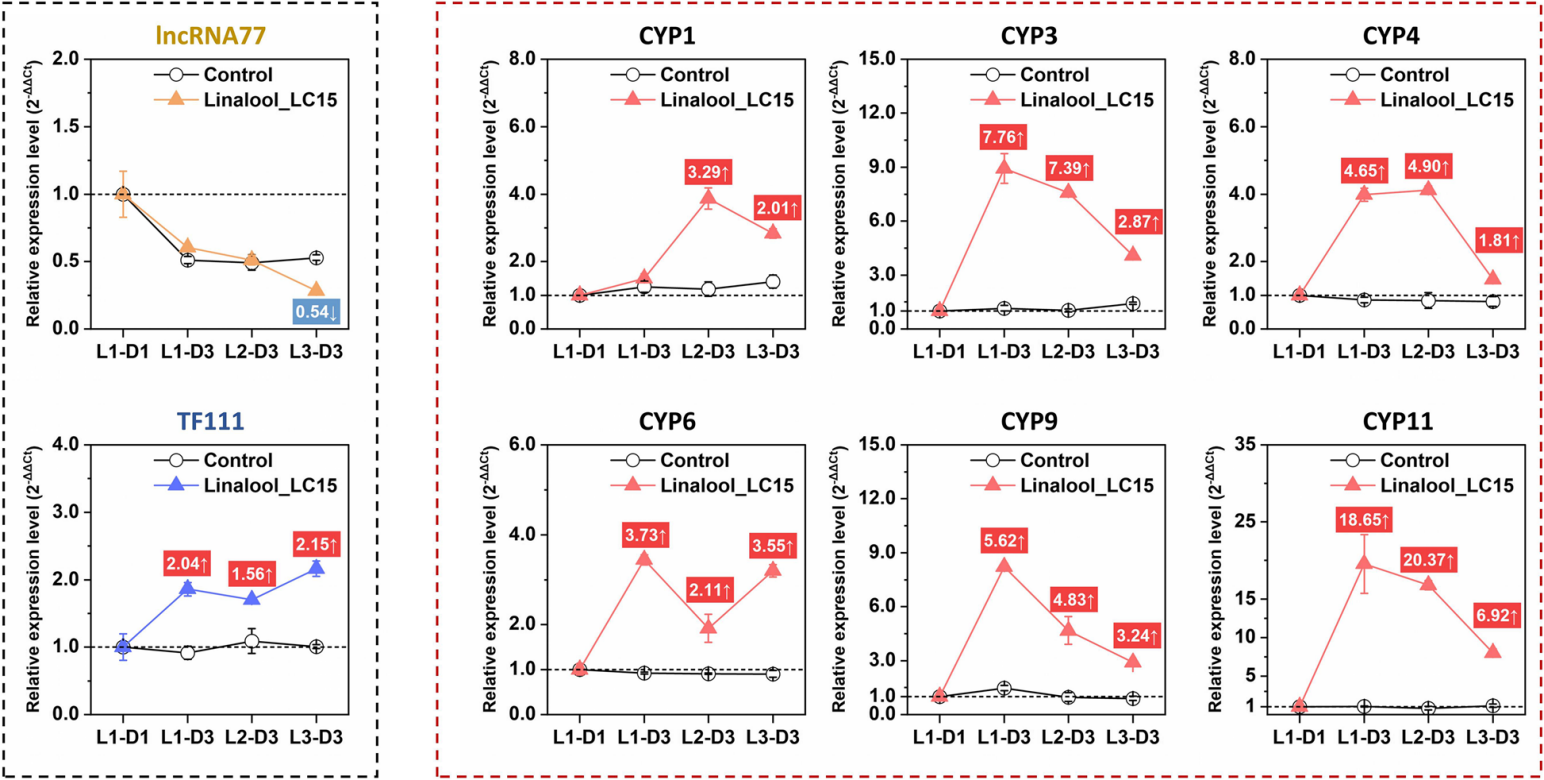
Temporal expression of the tested lncRNA, transcription factor, cytochrome P450 (CYP450) genes under Linalool exposure using RT-qPCR. Data are shown as the mean ± SE. The values with red or blue background indicate the fold changes between CK and Linalool_LC15 groups that reach a significant level according to one-way ANOVA followed by independent sample t-test (*P* < 0.05). L1-D1, day 1-1st instar larvae; L1-D3, day 3-1st instar larvae; L2-D3, day 3-2nd instar larvae; L3-D3, day 3-3rd instar larvae

**Fig. 6:**
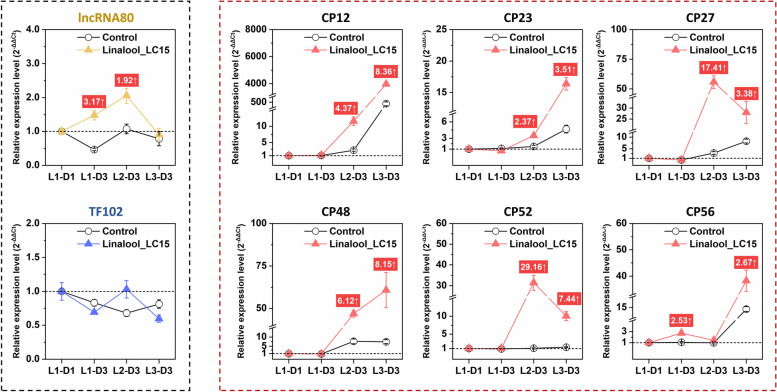
Temporal expression of the tested lncRNA, transcription factor, cuticular protein (CP) genes under Linalool exposure using RT-qPCR. Data are shown as the mean ± SE. The values with red or blue background indicate the fold changes between CK and Linalool_LC15 groups that reach a significant level according to one-way ANOVA followed by independent sample t-test (*P* < 0.05). L1-D1, day 1-1st instar larvae; L1-D3, day 3-1st instar larvae; L2-D3, day 3-2nd instar larvae; L3-D3, day 3-3rd instar larvae

To further investigate the roles of these candidate transcripts involving in response to dietary linalool, a long-lasting monitor on their expression levels from 1st- to 3rd instar larvae was performed. As shown in Fig. 5, lncRNA77 and TF111, as potential regulators of CYP450 transcription, showed a contradictory trend that the expression of the former continued to be suppressed with the increasing of larval instar, while the latter was significantly induced by linalool treatment, with 1.56- to 2.15-fold higher than the control. In regard to six CYP450 transcripts, varying degrees of up-regulation were observed when the larvae chronically fed on linalool-contained diets, with fold-change values ranging from 1.81 to 20.37. More interestingly, there was a bell-shaped relationship between the fold changes and developmental stages occurring in the majority of tested CYP450s. In addition, six transcripts encoding cuticular proteins showed a global up-regulation during the later stages of treatment (i.e. 2nd to 3rd instar), and the fold-change values varied from two to 30 (Fig. 6). lncRNA80, one hub lncRNA related to the expression of CPs, was slightly up-regulated. However, there is no significant changes occurring in the mRNA level of TF102 that was considered as one of hub TFs (Fig. 6).

## Discussion

### A global picture of *Pagiophloeus tsushimanus* transcriptome

To take advantage of RNA-seq and SMRT sequencing to offset the disadvantages of each alone, the combination of the two techniques has been increasingly applied to produce more complete transcripts [38, 39]. In this study, we adopted this hybrid-sequencing strategy to successfully construct the full-length transcriptome database of the weevil pest, *Pagiophloeus tsushimanus*. To be concise, we obtained 20,325 full-length transcripts with 1194 bp of average length and 1759 bp of N50 length. In other Coleoptera insects, transcriptome data of red palm weevil *Rhynchophorus ferrugineus* has been generated with a high quality in read length (average length 2964 bp and N50 length 3547 bp) [37], while *P. tsushimanus* transcriptome obtained in the present study shared a similar sequence length with the full-length transcriptome of flea beetle *Agasicles hygrophila* (average length 1684 bp and N50 length 2331 bp) [37]*.* We speculated that differences in read length were closely related to insect species and sequencing depth. In some studies, multiple cDNA libraries with different sizes (e.g. 1–2, 2–3, and 3–6 kb) were established, and using several SMRT cells to sequence these libraries [40, 41]. However, in this work, only one SMRT cell was used for one pooled cDNA library (1–6 kb in length), which results in a relatively short read length. Occurrence of alternative splicing (AS) events in the post-transcriptional stage increases the complexity and diversity of the transcriptome, and plays a role in response to environmental cues [42]. Herein, only 30 AS events were captured from *P. tsushimanus* transcriptome, while other information on AS events was not available due to a lack of reference genome. Simple sequence repeats (SSRs), referring to tandem repeats of one to six nucleotides, have been widely applied to analysis of genetic structure and diversity [43]. The abundance levels of different SSR repeat types usually vary among different insect species. In analogy to our results, mono-nucleotide repeat motif is dominating in most insects [44]. However, the largest abundance of tri-nucleotide repeat motif occurs in some other insects, such as *Tomicus yunnanensis* and *Anoplophora chinensis* [45, 46]. We also found tri-nucleotide repeat motif was the second largest abundance. One possibility is that tri-nucleotide rarely produces sliding mutations of the coding frame, and thus may be more stable than other repeat types in the protein-coding region.

Moreover, a very high percentage of *P. tsushimanus* transcriptome was similar to that of the mountain pine beetle, *Dendroctonus ponderosae* (Fig. S2), which implies that a closely linked genetic background allows them to survive in their harsh and terpenoid-rich host environment. Notably, high-quality genomes of *Dendroctonus* spp. recently published provide valuable references for interaction studies between the Curculionoidea superfamily and their host terpenoids [47, 48]. In this context, some rapidly expanded and positively selected genes related to metabolic capacity, including cytochrome P450 and glutathione S-transferase gene families, were thought to promote *Dendroctonus* adaptations to oleoresin terpenoids in conifers [48, 49]. Consequently, in the future, genome sequence of *P. tsushimanus* deserves in-depth studies to perform a comparative analysis with genomes of *Dendroctonus* spp. Despite the lack of genome resource, full-length *P. tsushimanus* transcriptome in this study facilitates the functional study and decipher mechanism underlying host adaptations.

**Transcriptional responses underlying the tolerance of** ***Pagiophloeus tsushimanus*** **larvae to dietary linalool.**

**Fig. 7:**
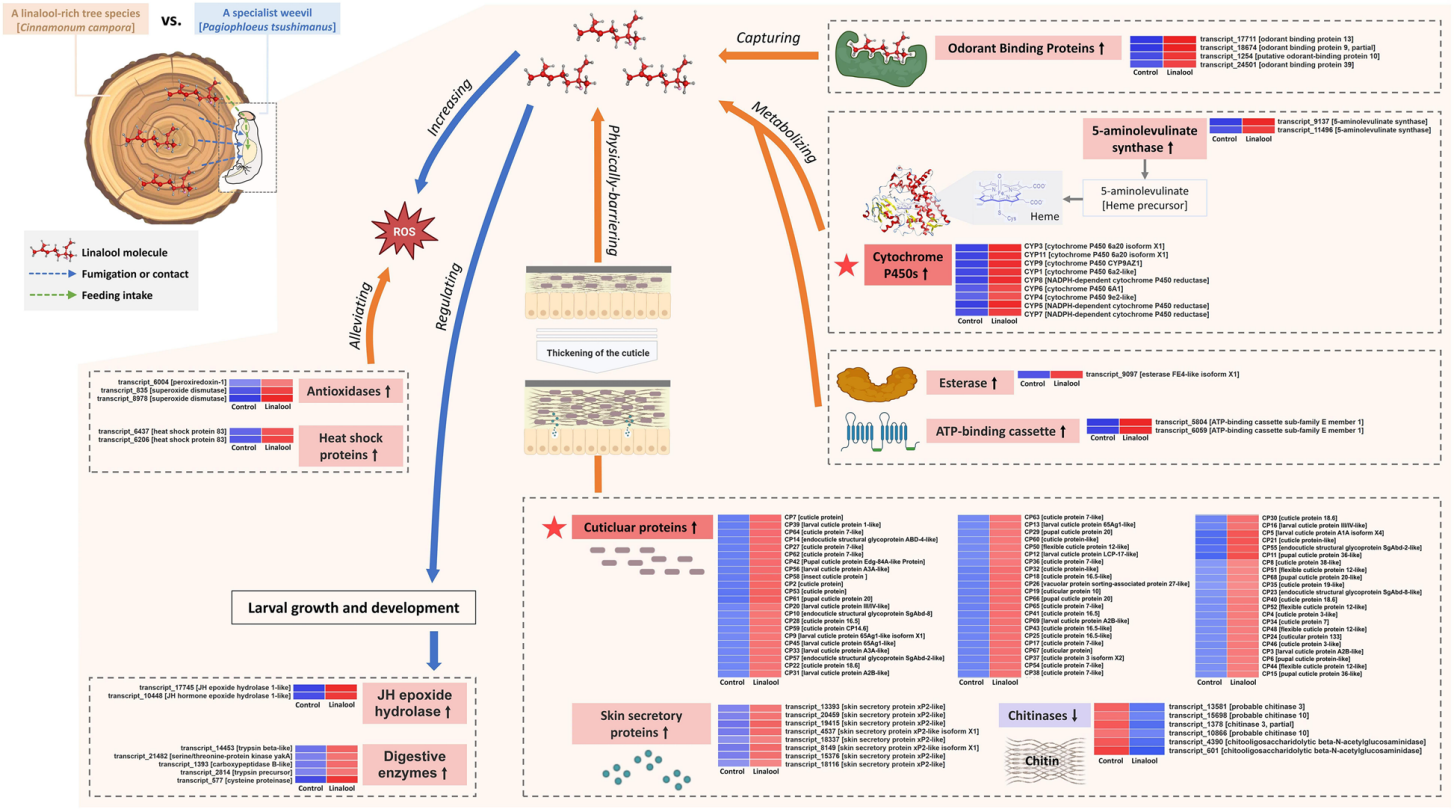
Proposed mode for mechanism underlying tolerance to dietary linalool primarily involves co-expression of cytochrome P450s (CYP450s) and cuticular proteins (CPs) in *Pagiophloeus tsushimanus* larvae

To understand the functional molecules recruited by *P. tsushimanus* larvae to counteract the specific xenobiotics of their unique host plant (i.e. a suit of monoterpenoid oxygenated derivatives from camphor trees), differential expression profile of the larvae upon exposure to dietary linalool were investigated in this study. The remarkable finding is that the pathways related to oxidoreductase activity and cuticular structural constituents were in an especially active stage, as characterized by a cluster of up-regulated transcripts encoding cytochrome P450s (CYP450s) and cuticular proteins (CPs) (Fig. 2). Other physiological effects possibly caused by linalool exposure, such as increase in Reactive Oxygen Species (ROS) and hormetic stimulation, were compensated by a handful of induced genes encoding antioxidases, heat shock proteins (HSPs), juvenile hormone (JH) epoxide hydrolases, and digestive enzymes (Fig. 2). Therefore we proposed a mode for mechanism underlying tolerance to dietary linalool primarily involves co-expression of CYP450s and CPs in *P. tsushimanus* larvae (Fig. 7).

From the perspective of detoxification metabolism, CYP450s as a key phase I detoxification enzyme is widely known to metabolize a variety of allelochemicals via its monooxygenation reaction [50]. Ample evidence has accumulated in the last few years indicating the induction of CYP450s by specific phytochemicals [51]. Our present results confirm that linalool is a phytochemical inducer of CYP450s in *P. tsushimanus* larvae. It is presumed that the biological activity of linalool against the tested larvae may be predominantly reduced by the oxidation of CYP6A subfamily. It is of valuable reference that well-established studies of CYP6 subfamily functioning in terpene detoxification have been conducted in bark beetles [18]. In these reports, a number of functionally-characterized CYP450s have been thought to hydroxylate the terpenes in conifers to some alcohol products as pheromone precursors or kairomones [17, 52–54]. However, the metabolic fate of some terpenoid derivatives from broad-leaved tree species (e.g. D-camphor and linalool from camphor trees) in insects remains poorly understood. Similarly, a bank of CYP450 transcripts in our former investigations were up-regulated after D-camphor exposure [55]. We expected that there is a parallel CYP450-mediated metabolic process occurring in the detoxification of D-camphor and linalool since they are a group of isomers. Furthermore, it is alarming that to what extent the induction of CYP450s represents their metabolic functions is still elusive. Thus we will soon further decipher the biological roles of these inducible CYP450s in the metabolism of host-specific terpenoids using the heterologous expression of insect P450 enzymes and RNAi experiments [56–58]. Besides, overexpression of 5-aminolevulinate synthases indicated that production of 5-aminolevulinate, as the precursor of an indispensable cofactor (called heme), assists in the metabolic reaction of CYP450s [59]. We also found that a fraction of up-regulated odorant binding proteins (OBPs) potentially capture the linalool molecule as signals to activate CYP450s or other downstream responses.

Unlike other chemicals, terpenoids, particularly mono- and sesquiterpenes, used in most studies belong to a class of plant volatiles that can be ingested by contact with the cuticle or fumigation [10]. The insect cuticle is the first and major barrier to prevent the penetration of exogenous compounds [60]. In the current study we revealed a great accumulation of transcripts coding for structural cuticular proteins (CPs) and skin secretory proteins in the treated larvae, suggesting their role in decreasing the penetration of linalool. Consistently, some of transcriptomic studies on the insects’ adaptation towards host plants also identified the differential expression of cuticle-related genes [61]. Meanwhile, the down-regulation of chitinase-related genes was found in the treated larvae. We speculate this negative regulation allows excessive chitin to be accumulated to achieve thickening of the cuticle.

Another is that CP family also participates in the developmental process of insect molting and metamorphosis, and consequently is sensitive to hormonal regulations [62]. Insect pheromones control the production of cuticle formation and mating behaviour. As found by Sureshan et al. [63], a number of genes related to pheromone biosynthesis and CP genes were differentially expressed when *Oxycarenus laetus* fed on gossypol soaked cotton seeds. That is reasonable to hypothesize that gossypol may be directly or indirectly involved in the excessive pheromone production, leading to the enhanced cuticle production in *O. laetus*. In the present work, two genes encoding JH epoxide hydrolases correspondingly exhibited a significant upregulation, suggesting that the ingestion of exogenous linalool may also directly or indirectly stimulate the excessive pheromone production, resulting ultimately in the differential response of CP family. To our knowledge, terpenes are widely considered as a precursor for the pheromone system of bark beetles [64], and JH induction for pheromone synthesis among bark beetles is well documented [53, 54, 65]. Therefore this potential cascade caused by the hormetic stimulation merits further investigation.

Finally, the intake of linalool may also affect digestive process of nutrients from the host plant, as characterized by up-regulation of some genes encoding digestive enzymes, and consequently regulate the larval growth and development. A similar case has shown that low dose of gossypol acts as a feeding stimulus to promote the growth of *Helicoverpa armigera* larvae by increasing food consumption rate [66]. More evolutionary explanation for these beneficial effects of specific plant defensive compounds on the specialist herbivores remain to further investigate. Conversely, the detrimental effects of phytochemicals, typically as enhanced ROS production, pose a great challenge to insects survivorship [67, 68]. Herein, we also observed the enhanced expression of several antioxidase and HSP genes, which may play key roles in regulating the intracellular ROS balance and preventing protein denaturation to alleviate oxidative damage [69, 70].

## Conclusion

There is a long-standing consensus in plant-insect interactions that specialist insects possess more specific and sometimes more efficient detoxification systems when compared to generalist ones [14, 71, 72]. In this context, the evolution of specific tolerance mechanisms propelled by selective pressure of host chemicals has been witnessed in many examples [73, 74]. In our previous work, an emerging specialist pest, *Pagiophloeus tsushimanus*, feeding exclusively on camphor trees *Cinnamomum camphora*, has been reported that its tolerance performance on different host monoterpenoids including D-camphor and linalool varied [26]. Furthermore, a potential mechanism underlying the tolerance of this specialist to D-camphor exposure has been proposed [55]. In this work, we obtained a more comprehensive transcriptome of *P. tsushimanus* using a combination of RNA-seq and SMRT sequencing, and emphasized the functional transcripts involved in response to dietary linalool. Based on 20,325 identified high-quality full-length transcripts, a narrow, targeted set of differentially expressed (DE) transcripts predominantly consisting of cytochrome P450 (CYP450) and cuticular protein (CP) families were considered to perform important physiological functions in overcoming the pressure of host-specific chemical (i.e. linalool). According to the results of co-expression networks, some DE lncRNAs and transcription factors strongly associated with the expressions of CYP450 and CP families were respectively determined as key regulators to activate the transcriptional responses of the two types of target mRNAs. Likewise, we further monitored the transcription dynamics of candidate CYP450 and CP transcripts under the chronical exposure of dietary linalool, suggesting that the specific adaptation to linalool is embodied in a rapid and moderate induction of CYP450 transcripts and a cumulative and massive induction of CP transcripts. Overall, the combined findings of this study will provide a new idea for future works on the adaptation traits of specialists responding to their specific host chemicals, and also facilitate better pest management.

## Material and methods

### Insect culture, plant material and reagents

A laboratory colony of *Pagiophloeus tsushimanus* was established from adults in *Cinnamomum camphora* plantations in Songjiang district, Shanghai city, China (30°56′6.15″N, 121°12′32.76″E) in May 2021. A complete breeding system for *P. tsushimanus* in the laboratory was described in our previous study [25]. In brief, the adults were allowed to feed, mate randomly and oviposit on fresh branches of camphor tree in a plastic cage. The eggs were kept in a Petri dish (5 cm i.d. × 1 cm) with moist cotton for incubation. The neonate larvae were maintained into a six-well Petri dish (3.5 cm i.d. × 1.5 cm each well) with fresh semi-artificial diets (50% distilled water, 40% powder of camphor tree bark, 5% agar, 3% sucrose, 1% yeast, 0.5% sorbic acid and 0.5% sodium benzoate) until pupation. The pupae were collected and deposited in glass tubes (2.0 cm i.d. × 5.0 cm) for emergence. All insects were kept in an environmental incubator (MTI-201B, Tokyo Rikakikai, Japan) at 28 ± 1 °C with a 70 ± 5% relative humidity and a photoperiod of 16: 8 h (L: D). Finally, the plant material authorized and used in insect culture was collected from the campus of Nanjing Forestry University (32°4′43″N/118°48′30″E), China, and authenticated by Prof. Zengfang Yin from Nanjing Forestry University, China. The voucher specimen (NF2002553) was deposited at Dendrological Herbarium, Nanjing Forestry University (Institution code from Chinese Virtual Herbarium: NF). All reagents for semi-artificial diets were purchased from Sangon, Shanghai, China. Linalool (95% purity, CAS: 78–70-6) was purchased from Sigma-Aldrich, Shanghai, China.

### Sample preparation and RNA extraction

**Fig. 8:**
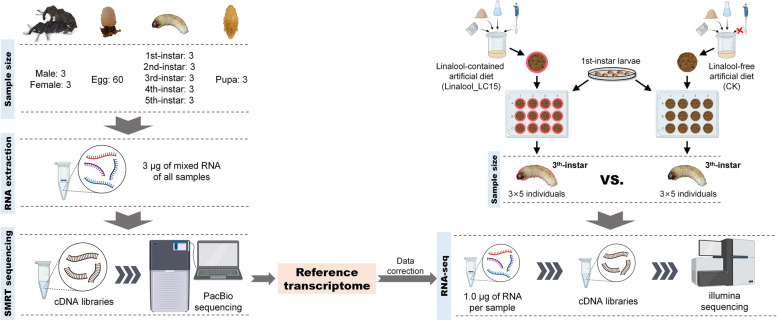
Experimental procedure for dietary linalool treatments, sampling, and sequencing

The procedure of sample preparation for SMRT sequencing and Illumina RNA-seq was visualized in Fig. 8.

For SMRT sequencing, three newly emerged males and females, sixty newly laid eggs, three newly molted 1st- to 5th-instar larvae, and three pupae aged 1–3 days were collected and frozen in liquid nitrogen for RNA extraction. Total RNA of each SMRT sequencing sample was respectively isolated using TRIzol Reagent (TaKaRa, Japan) according to the manufacturer’s protocol. RNA quantity was checked using a Nanodrop 2000 (Thermo Scientific, Waltham, MA, United States). RNA integrity was monitored on 1% agarose gels. An equal amount of total RNA from each sample was mixed in order to increase the sequence coverage of SMRT sequencing.

For Illumina RNA-seq, the neonate larvae were separated into two experimental groups. On the one hand larvae in the treatment group were reared on linalool-contained diets at the lethal concentration killing 15% of the population (19.7 mg·g^− 1^) (unpublished data) until they reached the 3th-instar stage (approximately 20 days old) (group name: Linalool_LC15), on the other larvae in the control group were reared on linalool-free diets until they reached the 3th-instar stage (group name: CK). Vigorous larvae from each group were selected and frozen in liquid nitrogen for subsequent tests. Three biological replicates with five larvae per replicate (3 × 5 individuals) were used for RNA extraction. Total RNA of each replicate was isolated and checked as described above. Six RNA samples (2 groups × 3 replicated samples) were used for Illumina RNA-seq.

### Library construction and sequencing

For SMRT sequencing, a mixed RNA sample (3 μg) as described above was used to generate a Full-length cDNA library according to the manufacturer’s protocol of SMARTerTM PCR cDNA Synthesis Kits (Clontech, USA). One SMRT cell (1–6 kb) was sequenced using a PacBio Sequel platform (Majorbio, Shanghai, China). The raw SMRT data were uploaded to the Genome Sequence Archive (GSA) (https://ngdc.cncb.ac.cn/gsa/) with accession CRA007259.

For Illumina RNA-seq, six cDNA libraries (2 groups × 3 replicated samples; 1 μg of RNA used per sample) were generated with NEBNext UltraTM RNA Library Prep Kits (NEB, Beverly, MA, United States) following the manufacturer’s protocol. The high quality of the libraries was confirmed using a high-sensitivity DNA chip on an Agilent Bioanalyzer 2100 (Agilent Technologies, Palo Alto, CA, United States). Paired-end sequencing was conducted on the Illumina HiSeq 2000 platform (Majorbio, Shanghai, China). The raw Illumina data were uploaded to the Genome Sequence Archive (GSA) (https://ngdc.cncb.ac.cn/gsa/) with accession CRA007253.

### Processing of SMRT sequencing data and Illumina data

To obtain the clean reads, the raw SMRT data were polished via filtering out short length (< 50 bp) and low quality (< 0.9) of polymerase reads using SMRTlink version 6.0 software. Then, a circular consensus sequence (CCS) was generated after the clean reads were processed into error-corrected reads of inserts (ROIs) with parameters of full passes of ≥3 and accuracy of > 0.9, and CCS.BAM files were output. CCS.BAM files were divided into full-length non-chimeric (FLNC) and non-full-length (NFL) reads according to whether the reads contained the 5′ primer, 3′ primer, and poly A tail. Subsequently, to obtain polished consensus isoforms, FLNC reads were clustered using the Iterative clustering of error correction (ICE) program and NFL reads were clustered using Quiver algorithm. High-quality isoforms with a post-correction accuracy of > 99% were identified as full-length transcripts. Ultimately, the final full-length transcripts were generated for further analysis after removing the redundancies by CD-HIT-EST program with default parameters.

The raw Illumina data were filtered by removing reads containing adapters, > 10% ambiguous ‘N’ nucleotides, and > 20% of the bases having quality scores < 10 in SeqPrep software (https://github.com/jstjohn/SeqPrep). The Q20, Q30 and GC content of the clean reads were calculated to assess sequencing quality using fastx_toolkit_0.0.14 (http://hannonlab.cshl.edu/fastx_toolkit/). The obtained full-length transcripts from SMRT data were applied as a reference transcriptome to Illumina data correction. We used Bowtie2 version 2.2.9 to map the clean reads from Illumina sequencing to the reference transcriptome with default parameters [75].

### Prediction of alternative splicing, simple sequence repeat, long non-coding RNA, and transcription factor

Alternative splicing (AS) events were predicted using pair-wise BLAST analysis based on the full-length transcript sequences. The alignment results were considered as candidate AS events according to the following criteria: 1) Both tested sequences lengths were more than 1000 bp, and there should be two High-scoring Segment Pairs in the alignment. 2) The alternative splicing gap was more than 100 bp, with at least 100 bp distance to the 3′/5′ end. 3) All alternatively-spliced transcripts allowed 5 bp overlap.

Transcripts > 500 bp were subjected to simple sequence repeat (SSR) analysis using MISA version 2.3.6. Seven SSR types, including mononucleotide, dinucleotide, trinucleotide, tetra-nucleotide, pentanucleotide, hexanucleotide, and compound SSRs (mixed microsatellites, two SSRs with > 100 bp distance), were detected.

long non-coding RNA (lncRNA) was predicted using Coding Potential Calculator (CPC, score < 0.5), Coding-Non-Coding Index (CNCI, score < 0), PLEK (score < 0), and Pfam protein structure domain analysis (no annotation). The prediction of transcription factors (TFs) was performed by the animal TFDB version 3.0 database.

### Functional annotation of full-length transcripts

To annotate the obtained full-length transcripts, six public databases, namely, NCBI nonredundant proteins (Nr, e-value ≤1.0 × 10^− 5^), Swiss-Prot (e-value ≤1.0 × 10^− 5^), Protein families (Pfam, e-value ≤1.0 × 10^− 5^), Clusters of Orthologous Groups (COG, e-value ≤1.0 × 10^− 5^), (Gene Ontology (GO, e-value ≤1.0 × 10^− 5^), and Kyoto Encyclopedia of Genes and Genomes (KEGG, e-value ≤1.0 × 10^− 10^) databases, were searched [76].

### Differential expression analysis and GO enrichment analysis of full-length transcripts

The expression level of each transcript from six RNA-seq samples was calculated and normalized as TPM (transcripts per million) values by RSEM version 1.3.1. Then, we used DESeq2 version 1.24.0 for differential expression analyses between CK and Linalool_LC15 groups. The criteria for determining the differentially expressed (DE) transcripts was *P*-value < 0.05 (false discovery rate ≤ 0.01) and |log_2_ (fold change)| ≥ 1. For significantly up-regulated and down-regulated transcripts, GO enrichment analysis was separately performed using Goatools program (https://github.com/tanghaibao/GOatools), and an adjusted *P*-value < 0.05 was the threshold value for determining the significantly enriched terms. Heatmap of differentially expressed transcripts of interest were present using Chiplot online tools (https://www.chiplot.online/tvbot.html).

### lncRNA-mRNA and TF-mRNA co-expression networks construction

According to the results of differential expression analysis and GO enrichment analysis, the DE transcripts encoding cytochrome P450s (CYP450s) and cuticular protein (CPs) were identified as target genes (mRNAs) that involved in tolerance to dietary linalool. To screen for the regulating factors associated with target mRNAs, the correlation coefficients between target mRNAs and lncRNAs, target mRNAs and TF were evaluated on the basis of TBM value using the Pearson correlation method in Origin version 2021b. The correlation coefficients of > 0.8 (positive) or < 0.8 (negative) between target mRNAs and lncRNAs, target mRNAs and TF were considered as co-expressed genes. Data of correlation analysis were put into Cytoscape 3.8.2 to generate co-expression networks.

### Expression pattern of interesting transcripts using real-time quantitative PCR

We selected two lncRNAs, two TFs, six CYP450 transcripts, and six CP transcripts to explore their transcriptional responses to linalool exposure using Real-time quantitative PCR (RT-qPCR). The sampling scheme was as follows: Day 1, 1st instar larvae (L1-D1), Day 3, 1st instar larvae (L1-D3), Day 3, 2nd instar larvae (L2-D3), and Day 3, 3rd instar larvae (L3-D3) were respectively collected from the treatment and CK groups referring to the protocols for Illumina RNA-Seq samples. Three biological replicates with five larvae per replicate (3 × 5 individuals) were used for each time point. All samples were frozen in liquid nitrogen for RNA extraction. cDNA was synthesized using a HiScript 1st Strand cDNA Synthesis Kit according to the manufacturer’s protocol (Vazyme Biotech, Nanjing, China). RT-qPCR experiments were performed using SYBR Premix Ex Taq II (TaKaRa, Japan) on an Applied Biosystem 7500 Real-Time PCR System (Thermo Fisher, Massachusetts, United States). PCR conditions were as follows: 5 min at 95 °C, followed by 40 cycles of 10 s at 95 °C and 40 s at 60 °C. Specific primers were designed using Primer Premier version 5.0 (Table S8) and synthesized from Sangon, Shanghai, China. Relative expression levels of each transcript were determined by the 2^−△△Ct^ method with Ribosomal protein L10 (RPL10) and TBP (TATA-binding protein) as the housekeeping genes. Statistical significance of gene expression was determined using one-way ANOVA followed by independent sample *t*-test (*P* < 0.05) in SPSS version 20.0. To verify the reliability of RNA-Seq data, Pearson’s correlation between the results of RT-qPCR (Log2 2^−△△Ct^) and RNA-Seq (Log2 fold change) was analysed in SPSS version 20.0.

## Supplementary Information

**Additional file 1.**

## Data Availability

The raw SMRT data generated during the current study were uploaded to the Genome Sequence Archive (GSA) (https://ngdc.cncb.ac.cn/gsa/) with accession CRA007259. The raw Illumina data generated during the current study were uploaded to the Genome Sequence Archive (GSA) (https://ngdc.cncb.ac.cn/gsa/) with accession CRA007253. The qPCR data were uploaded to figshare (https://figshare.com/account/home). Li, Shouyin (2022): Data for qPCR. figshare. Dataset. 10.6084/m9.figshare.20941435
