# Supplementary material for: Tolerance to dietary linalool primarily involves co-expression of cytochrome P450s and cuticular proteins in Pagiophloeus tsushimanus (Coleoptera: Curculionidae) larvae using SMRT sequencing and RNA-seq
Source: BMC Genomics. 2023 Jan 19;24:34. doi: 10.1186/s12864-023-09117-7 (PMC9854079; doi:10.1186/s12864-023-09117-7)
Supplement: Supplementary file 1 — Additional file 1. [file 12864_2023_9117_MOESM1_ESM.docx]

**Table S1. Summary of the transcriptome data from PacBio Sequel platform**

| Platform | PacBio |
| --- | --- |
| SMRT cell number | 1 |
| cDNA library size | 1-6 K |
| Subreads base (G) | 18.31 |
| Number of subreads | 16,263,362 |
| CCS base (bp)^1^ | 203,387,693 |
| Number of CSS | 113,436 |
| CCS mean length (bp) | 1792.97 |
| CCS N50^2^ | 28036 |
| CCS GC content (%)^3^ | 21.836 |
| FLNC base (bp)^4^ | 61,526,602 |
| Number of FLNC | 68,407 |
| FLNC mean length (bp) | 899.42 |
| FLNC N50 | 7633 |
| FLNC GC content (%) | 40.277 |
| Consensus base (bp) | 29,482,566 |
| Number of consensus | 26,937 |
| Consensus mean length (bp) | 1094.50 |
| Consensus N50 | 7633 |
| Consensus GC content (%) | 39.379 |
| FL transcript base (bp) | 24,274,424 |
| Number of FL transcript | 20,325 |
| FL transcript mean length (bp) | 1194.31 |
| Transcript N50 | 1759 |
| Transcript GC content (%) | 39.22 |

^1^ CCS, circular consensus sequences

^2^ N50, the sequence length at 50% of the total transcript length.

^3^ GC content, proportion of G and C bases

^4^ FLNC, full-length non-chimera

**Table S2. Summary of the transcriptome data from Illumina HiSeq 2000 platform**

| **Group name** | **Sample name** | **Raw reads** | **Raw bases**  **(Gb)** | **Clean reads** | **Clean bases (Gb)** | **Error rate**  **(%)** | **Q20^1^ (%)** | **Q30^2^**  **(%)** | **GC^3^**  **(%)** |
| --- | --- | --- | --- | --- | --- | --- | --- | --- | --- |
| CK | A1 | 49,150,654 | 7.42 | 48,846,670 | 7.21 | 0.0238 | 98.51 | 95.34 | 43.42 |
|  | A2 | 49,569,952 | 7.49 | 49,298,878 | 7.27 | 0.0236 | 98.61 | 95.61 | 43.65 |
|  | A3 | 49,667,838 | 7.50 | 49,384,444 | 7.30 | 0.0235 | 98.64 | 95.68 | 43.47 |
| Linalool_LC15 | B1 | 50,574,016 | 7.64 | 50,298,196 | 7.45 | 0.0236 | 98.62 | 95.59 | 42.61 |
|  | B2 | 51,258,908 | 7.74 | 50,936,068 | 7.52 | 0.0236 | 98.60 | 95.55 | 43.13 |
|  | B3 | 53,143,406 | 8.02 | 52,831,644 | 7.83 | 0.0236 | 98.61 | 95.60 | 42.93 |
| Average | | 50,560,795 | 7.64 | 50,265,983 | 7.43 | 0.0236 | 98.60 | 95.56 | 43.20 |

^1^ Q20, percentage of bases with quality over a Phred score of 20 (i.e., an error rate of 1 %)

^2^ Q30, percentage of bases with quality over a Phred score of 30 (i.e., an error rate of 0.1 %)

^3^ GC, proportion of G and C bases

**Table S3. Distribution of Single Sequence Repeats (SSRs) according to the number of repeat motifs**

| **SSR motif unit** | **SSR type** | **Repeat number** | | | | **Total** | **Percentage (%)** |
| --- | --- | --- | --- | --- | --- | --- | --- |
|  |  | **1-5** | **6-10** | **11-15** | **>15** |  |  |
| Mono-nucleotide  motifs | A/T | 0 | 2,743 | 2,472 | 825 | 7,201 | 89.46 |
|  | C/G | 0 | 152 | 381 | 628 |  |  |
| Di-nucleotide  motifs | AC/GT | 0 | 60 | 6 | 1 | 265 | 3.29 |
|  | AG/CT | 0 | 32 | 3 | 0 |  |  |
|  | AT/AT | 0 | 133 | 5 | 1 |  |  |
|  | CG/CG | 0 | 24 | 0 | 0 |  |  |
| Tri-nucleotide  motifs | AAC/GTT | 37 | 4 | 0 | 0 | 551 | 6.85 |
|  | AAG/CTT | 90 | 4 | 0 | 0 |  |  |
|  | AAT/ATT | 95 | 87 | 3 | 1 |  |  |
|  | ACC/GGT | 31 | 14 | 0 | 0 |  |  |
|  | ACG/CGT | 9 | 9 | 1 | 0 |  |  |
|  | ACT/AGT | 15 | 2 | 0 | 0 |  |  |
|  | AGC/CTG | 16 | 13 | 0 | 1 |  |  |
|  | AGG/CCT | 35 | 16 | 0 | 0 |  |  |
|  | ATC/ATG | 15 | 14 | 0 | 0 |  |  |
|  | CCG/CGG | 35 | 4 | 0 | 0 |  |  |
| Tetra-nucleotide  motifs | AAAC/GTTT | 0 | 4 | 0 | 0 | 28 | 0.35 |
|  | AAAG/CTTT | 1 | 0 | 0 | 0 |  |  |
|  | AAAT/ATTT | 14 | 3 | 0 | 0 |  |  |
|  | AATC/ATTG | 2 | 0 | 0 | 0 |  |  |
|  | AATT/AATT | 1 | 0 | 0 | 0 |  |  |
|  | AGAT/ATCT | 1 | 0 | 1 | 0 |  |  |
|  | ATCC/ATGG | 1 | 0 | 0 | 0 |  |  |
| Penta-nucleotide  motifs | AAAAT/ATTTT | 1 | 0 | 0 | 0 | 4 | 0.05 |
|  | AAATC/ATTTG | 0 | 2 | 0 | 0 |  |  |
|  | AATAT/ATATT | 1 | 0 | 0 | 0 |  |  |
| Total |  | 400 | 3,320 | 2,872 | 1,457 | 8,049 | 100.00 |
| Percentage (%) |  | 4.97 | 41.25 | 35.68 | 18.10 |  |  |

**Table S4. Annotation statistics of the assembled RNA-seq data**

| **Database** | **Number of transcript** | **Ratio (%)** |
| --- | --- | --- |
| COG | 11,368 | 55.93 |
| GO | 8,623 | 42.43 |
| KEGG | 12,305 | 60.54 |
| NR | 14,266 | 70.19 |
| Swiss-Prot | 10,105 | 49.72 |
| Pfam | 10,539 | 51.85 |
| Total_anno^1^ | 14,492 | 71.30 |
| Total | 20,325 | 100.00 |

^1^ Total_anno, transcripts that had matches in at least one database.

**Table S5. Expression levels of differentially expressed transcripts of interest**

| **Gene name** | **Gene_id** | **Transcripts Per Million (TPM)** | | | | | | | |
| --- | --- | --- | --- | --- | --- | --- | --- | --- | --- |
|  |  | **Linalool_LC15** | | | | **CK** | | | |
|  |  | **B3** | **B2** | **B1** | **Average** | **A3** | **A2** | **A1** | **Average** |
| CP1 | transcript_1063 | 0.0 | 0.3 | 0.0 | 0.1 | 0.7 | 3.7 | 3.8 | 2.7 |
| CP2 | transcript_10960 | 2.5 | 9.8 | 23.3 | 11.9 | 0.3 | 0.6 | 0.7 | 0.5 |
| CP3 | transcript_1216 | 7.9 | 71.1 | 279.5 | 119.5 | 0.1 | 0.0 | 0.0 | 0.0 |
| CP4 | transcript_12532 | 0.5 | 4.3 | 16.9 | 7.2 | 0.1 | 0.3 | 0.0 | 0.1 |
| CP5 | transcript_12778 | 0.8 | 2.0 | 4.0 | 2.3 | 0.1 | 0.2 | 0.0 | 0.1 |
| CP6 | transcript_12963 | 0.1 | 0.2 | 1.4 | 0.6 | 0.0 | 0.0 | 0.0 | 0.0 |
| CP7 | transcript_14050 | 1.5 | 2.2 | 6.8 | 3.5 | 0.6 | 0.5 | 0.8 | 0.6 |
| CP8 | transcript_1406 | 0.3 | 0.6 | 6.0 | 2.3 | 0.0 | 0.0 | 0.0 | 0.0 |
| CP9 | transcript_14089 | 5.5 | 14.3 | 45.4 | 21.7 | 2.2 | 2.1 | 4.9 | 3.1 |
| CP10 | transcript_14227 | 6.5 | 35.2 | 122.6 | 54.8 | 0.6 | 0.7 | 0.7 | 0.7 |
| CP11 | transcript_14243 | 0.1 | 0.8 | 0.9 | 0.6 | 0.0 | 0.0 | 0.0 | 0.0 |
| CP12 | transcript_1427 | 27.7 | 156.7 | 598.9 | 261.1 | 3.9 | 1.6 | 1.8 | 2.4 |
| CP13 | transcript_14681 | 11.7 | 80.2 | 285.1 | 125.7 | 2.3 | 0.0 | 3.3 | 1.9 |
| CP14 | transcript_15186 | 3.0 | 11.6 | 34.2 | 16.3 | 0.3 | 0.1 | 0.0 | 0.1 |
| CP15 | transcript_15389 | 1.0 | 3.5 | 19.0 | 7.8 | 0.0 | 0.0 | 0.0 | 0.0 |
| CP16 | transcript_1540 | 0.7 | 4.5 | 14.2 | 6.5 | 0.0 | 0.0 | 0.0 | 0.0 |
| CP17 | transcript_1565 | 33.1 | 121.4 | 422.4 | 192.3 | 1.2 | 1.4 | 0.4 | 1.0 |
| CP18 | transcript_15673 | 10.4 | 28.4 | 95.8 | 44.9 | 0.5 | 0.1 | 1.2 | 0.6 |
| CP19 | transcript_15693 | 19.4 | 36.0 | 115.3 | 56.9 | 7.1 | 1.6 | 11.5 | 6.7 |
| CP20 | transcript_1591 | 0.6 | 4.8 | 15.7 | 7.0 | 0.0 | 0.0 | 0.0 | 0.0 |
| CP21 | transcript_15924 | 2.0 | 6.3 | 12.0 | 6.8 | 0.0 | 0.0 | 0.0 | 0.0 |
| CP22 | transcript_16056 | 58.5 | 192.7 | 720.8 | 324.0 | 2.1 | 3.9 | 2.6 | 2.8 |
| CP23 | transcript_1627 | 274.1 | 1058.2 | 3734.1 | 1688.8 | 354.6 | 254.6 | 351.8 | 320.3 |
| CP24 | transcript_16360 | 3.1 | 13.2 | 63.9 | 26.7 | 0.0 | 0.0 | 0.1 | 0.0 |
| CP25 | transcript_16593 | 2.9 | 23.3 | 67.0 | 31.1 | 0.2 | 0.5 | 0.1 | 0.3 |
| CP26 | transcript_16838 | 15.5 | 31.0 | 111.3 | 52.6 | 2.8 | 0.1 | 3.6 | 2.2 |
| CP27 | transcript_17003 | 61.6 | 151.2 | 520.2 | 244.4 | 0.0 | 0.0 | 0.0 | 0.0 |
| CP28 | transcript_17256 | 0.3 | 7.3 | 21.0 | 9.6 | 0.1 | 0.0 | 0.0 | 0.0 |
| CP29 | transcript_17460 | 1.2 | 6.6 | 23.2 | 10.3 | 0.2 | 0.3 | 0.4 | 0.3 |
| CP30 | transcript_17638 | 18.0 | 68.2 | 232.2 | 106.1 | 0.7 | 3.4 | 0.6 | 1.6 |
| CP31 | transcript_17675 | 0.5 | 4.6 | 14.6 | 6.6 | 0.2 | 0.4 | 0.0 | 0.2 |
| CP32 | transcript_17740 | 2.5 | 6.0 | 21.8 | 10.1 | 0.0 | 0.0 | 0.0 | 0.0 |
| CP33 | transcript_17780 | 1.8 | 13.1 | 42.1 | 19.0 | 0.0 | 0.0 | 0.0 | 0.0 |
| CP34 | transcript_17937 | 1.2 | 4.1 | 15.3 | 6.9 | 0.4 | 0.9 | 0.7 | 0.7 |
| CP35 | transcript_1807 | 0.3 | 1.7 | 15.5 | 5.8 | 0.0 | 0.0 | 0.0 | 0.0 |
| CP36 | transcript_1819 | 52.1 | 117.0 | 587.6 | 252.2 | 0.2 | 1.2 | 0.6 | 0.7 |
| CP37 | transcript_18248 | 0.7 | 14.7 | 31.6 | 15.6 | 1.8 | 0.5 | 0.5 | 1.0 |
| CP38 | transcript_1865 | 53.3 | 151.1 | 574.1 | 259.5 | 1.5 | 2.4 | 1.5 | 1.8 |
| CP39 | transcript_18749 | 1.2 | 2.6 | 9.1 | 4.3 | 0.0 | 0.0 | 0.0 | 0.0 |
| CP40 | transcript_18808 | 0.8 | 2.9 | 14.2 | 5.9 | 0.0 | 0.0 | 0.0 | 0.0 |
| CP41 | transcript_1886 | 1.2 | 20.3 | 51.3 | 24.3 | 0.0 | 0.0 | 0.0 | 0.0 |
| CP42 | transcript_1967 | 2.1 | 25.2 | 58.2 | 28.5 | 0.0 | 0.4 | 0.0 | 0.1 |
| CP43 | transcript_19802 | 4.0 | 15.0 | 51.0 | 23.3 | 0.3 | 0.0 | 0.2 | 0.2 |
| CP44 | transcript_20047 | 109.2 | 263.9 | 886.5 | 419.8 | 152.1 | 57.7 | 42.0 | 83.9 |
| CP45 | transcript_20138 | 0.9 | 14.4 | 41.5 | 18.9 | 0.0 | 0.0 | 0.0 | 0.0 |
| CP46 | transcript_20223 | 21.7 | 124.0 | 503.7 | 216.5 | 2.6 | 1.8 | 2.8 | 2.4 |
| CP47 | transcript_20651 | 0.0 | 0.4 | 2.8 | 1.1 | 7.9 | 79.0 | 4.2 | 30.4 |
| CP48 | transcript_2117 | 109.3 | 766.5 | 3300.5 | 1392.1 | 22.2 | 9.7 | 24.8 | 18.9 |
| CP49 | transcript_21660 | 0.0 | 1.2 | 2.7 | 1.3 | 3.8 | 46.7 | 4.6 | 18.4 |
| CP50 | transcript_2170 | 217.3 | 661.6 | 2465.4 | 1114.8 | 60.6 | 55.8 | 132.4 | 82.9 |
| CP51 | transcript_22725 | 0.7 | 6.6 | 40.1 | 15.8 | 0.3 | 0.9 | 0.6 | 0.6 |
| CP52 | transcript_2403 | 58.7 | 444.6 | 1892.3 | 798.5 | 2.2 | 0.6 | 6.0 | 2.9 |
| CP53 | transcript_3080 | 11.7 | 17.2 | 48.6 | 25.8 | 3.4 | 1.3 | 4.6 | 3.1 |
| CP54 | transcript_330 | 28.1 | 77.1 | 293.0 | 132.7 | 0.2 | 3.7 | 0.4 | 1.4 |
| CP55 | transcript_410 | 1.7 | 9.0 | 15.0 | 8.6 | 0.0 | 0.1 | 0.7 | 0.3 |
| CP56 | transcript_413 | 17.3 | 83.5 | 228.3 | 109.7 | 1.6 | 1.3 | 2.0 | 1.6 |
| CP57 | transcript_417 | 67.9 | 117.8 | 339.6 | 175.1 | 46.7 | 32.4 | 46.7 | 41.9 |
| CP58 | transcript_4587 | 6.2 | 9.2 | 27.7 | 14.3 | 3.0 | 0.5 | 2.5 | 2.0 |
| CP59 | transcript_469 | 4.1 | 29.4 | 97.5 | 43.7 | 0.0 | 0.0 | 0.0 | 0.0 |
| CP60 | transcript_4866 | 5.2 | 10.8 | 40.8 | 18.9 | 2.3 | 1.4 | 3.3 | 2.4 |
| CP61 | transcript_4878 | 2.1 | 18.4 | 28.2 | 16.3 | 1.0 | 0.1 | 1.3 | 0.8 |
| CP62 | transcript_496 | 113.4 | 264.3 | 921.7 | 433.1 | 1.5 | 0.6 | 0.7 | 0.9 |
| CP63 | transcript_535 | 6.2 | 28.9 | 112.8 | 49.3 | 0.0 | 0.0 | 0.0 | 0.0 |
| CP64 | transcript_539 | 84.8 | 240.3 | 695.8 | 340.3 | 37.0 | 1.7 | 15.8 | 18.2 |
| CP65 | transcript_564 | 32.2 | 100.3 | 335.0 | 155.8 | 0.2 | 2.0 | 0.4 | 0.9 |
| CP66 | transcript_613 | 2.3 | 26.5 | 60.3 | 29.7 | 1.2 | 1.3 | 1.6 | 1.4 |
| CP67 | transcript_7264 | 0.5 | 2.0 | 4.9 | 2.4 | 0.5 | 0.2 | 0.2 | 0.3 |
| CP68 | transcript_761 | 0.1 | 1.2 | 7.6 | 3.0 | 0.0 | 0.0 | 0.0 | 0.0 |
| CP69 | transcript_909 | 13.3 | 122.7 | 330.2 | 155.4 | 6.9 | 1.6 | 2.5 | 3.7 |
| CYP1 | transcript_10275 | 534.2 | 575.8 | 291.3 | 467.1 | 89.1 | 107.2 | 59.8 | 85.4 |
| CYP2 | transcript_15627 | 340.3 | 452.1 | 358.8 | 383.7 | 1538.4 | 728.9 | 667.6 | 978.3 |
| CYP3 | transcript_17583 | 5690.2 | 5317.0 | 3055.7 | 4687.6 | 39.8 | 32.4 | 37.6 | 36.6 |
| CYP4 | transcript_22624 | 85.3 | 33.7 | 29.8 | 49.6 | 5.8 | 4.9 | 7.7 | 6.1 |
| CYP5 | transcript_3134 | 7.2 | 7.2 | 6.3 | 6.9 | 3.7 | 4.2 | 2.6 | 3.5 |
| CYP6 | transcript_4508 | 92.1 | 111.5 | 31.9 | 78.5 | 12.2 | 15.4 | 8.0 | 11.8 |
| CYP7 | transcript_7187 | 16.5 | 20.2 | 11.7 | 16.1 | 6.9 | 8.3 | 6.7 | 7.3 |
| CYP8 | transcript_8259 | 4.5 | 5.2 | 3.0 | 4.2 | 0.5 | 0.8 | 1.1 | 0.8 |
| CYP9 | transcript_9023 | 530.2 | 608.8 | 322.8 | 487.3 | 38.7 | 43.7 | 25.3 | 35.9 |
| CYP10 | transcript_9201 | 6.9 | 17.0 | 7.7 | 10.6 | 57.4 | 48.5 | 28.3 | 44.7 |
| CYP11 | transcript_9957 | 2875.2 | 2716.3 | 1376.6 | 2322.7 | 46.1 | 18.1 | 25.9 | 30.0 |
| - | transcript_13393 | 193.3 | 50.8 | 10.5 | 84.9 | 0.0 | 0.0 | 0.1 | 0.0 |
| - | transcript_20459 | 234.5 | 70.7 | 12.0 | 105.7 | 0.3 | 0.1 | 0.4 | 0.3 |
| - | transcript_19415 | 8.3 | 3.1 | 1.1 | 4.2 | 0.0 | 0.0 | 0.0 | 0.0 |
| - | transcript_4537 | 25.8 | 9.4 | 3.1 | 12.8 | 1.0 | 0.4 | 1.3 | 0.9 |
| - | transcript_18337 | 12.0 | 2.3 | 0.3 | 4.9 | 0.0 | 0.0 | 0.0 | 0.0 |
| - | transcript_8149 | 6.3 | 2.5 | 0.5 | 3.1 | 0.2 | 0.0 | 0.0 | 0.1 |
| - | transcript_15376 | 1.6 | 0.3 | 0.3 | 0.7 | 0.0 | 0.0 | 0.0 | 0.0 |
| - | transcript_18116 | 4.0 | 0.7 | 0.1 | 1.6 | 0.1 | 0.0 | 0.0 | 0.0 |
| - | transcript_14453 | 68.0 | 135.3 | 10.7 | 71.4 | 0.2 | 1.1 | 1.4 | 0.9 |
| - | transcript_21482 | 234.4 | 80.9 | 7.1 | 107.5 | 0.0 | 0.0 | 0.0 | 0.0 |
| - | transcript_1393 | 38.1 | 33.5 | 148.4 | 73.3 | 8.5 | 8.3 | 4.8 | 7.2 |
| - | transcript_2814 | 6.9 | 36.8 | 4.9 | 16.2 | 0.0 | 0.5 | 0.0 | 0.2 |
| - | transcript_577 | 202.9 | 141.7 | 163.8 | 169.5 | 88.1 | 64.1 | 99.1 | 83.8 |
| - | transcript_17711 | 7609.7 | 8560.5 | 9719.9 | 8630.0 | 28.6 | 66.3 | 89.6 | 61.5 |
| - | transcript_18674 | 351.0 | 297.5 | 302.6 | 317.0 | 89.1 | 84.2 | 68.2 | 80.5 |
| - | transcript_1254 | 611.8 | 1012.7 | 1257.7 | 960.7 | 187.2 | 251.5 | 198.9 | 212.5 |
| - | transcript_24501 | 21.2 | 40.2 | 61.0 | 40.8 | 6.1 | 3.5 | 6.8 | 5.5 |
| - | transcript_6004 | 26.2 | 18.1 | 20.8 | 21.7 | 3.7 | 5.2 | 4.2 | 4.4 |
| - | transcript_835 | 15.1 | 2.6 | 1.4 | 6.4 | 0.3 | 0.0 | 0.3 | 0.2 |
| - | transcript_8978 | 3.1 | 1.6 | 2.1 | 2.3 | 0.9 | 0.6 | 1.1 | 0.8 |
| - | transcript_9137 | 94.8 | 101.4 | 127.2 | 107.8 | 30.9 | 24.6 | 40.4 | 32.0 |
| - | transcript_11496 | 75.2 | 145.8 | 146.5 | 122.5 | 30.8 | 38.5 | 55.8 | 41.7 |
| - | transcript_5804 | 15.0 | 13.9 | 13.8 | 14.2 | 6.4 | 4.5 | 2.0 | 4.3 |
| - | transcript_6059 | 15.3 | 12.7 | 12.2 | 13.4 | 7.7 | 3.5 | 4.5 | 5.2 |
| - | transcript_6437 | 8.8 | 6.5 | 3.6 | 6.3 | 1.5 | 3.4 | 2.6 | 2.5 |
| - | transcript_6206 | 73.4 | 56.5 | 37.7 | 55.9 | 9.5 | 20.1 | 31.7 | 20.4 |
| - | transcript_17745 | 189.2 | 279.3 | 307.1 | 258.5 | 55.4 | 55.9 | 62.9 | 58.0 |
| - | transcript_10448 | 408.7 | 599.3 | 723.2 | 577.1 | 185.1 | 254.7 | 235.2 | 225.0 |
| - | transcript_9097 | 203.4 | 155.7 | 274.3 | 211.1 | 72.1 | 61.4 | 123.1 | 85.5 |
| - | transcript_13581 | 6.6 | 6.8 | 3.6 | 5.6 | 9.5 | 43.2 | 22.6 | 25.1 |
| - | transcript_15698 | 4.7 | 5.7 | 3.0 | 4.5 | 8.2 | 40.7 | 13.3 | 20.7 |
| - | transcript_1378 | 3.1 | 5.1 | 2.2 | 3.4 | 7.7 | 22.0 | 10.4 | 13.4 |
| - | transcript_10866 | 4.1 | 4.8 | 3.8 | 4.2 | 8.1 | 31.3 | 9.4 | 16.3 |
| - | transcript_4390 | 26.2 | 25.4 | 15.8 | 22.5 | 35.0 | 88.5 | 52.7 | 58.7 |
| - | transcript_601 | 10.8 | 7.0 | 8.7 | 8.9 | 15.1 | 26.4 | 18.6 | 20.0 |
| - | transcript_17514 | 186.4 | 102.0 | 217.5 | 168.6 | 3.8 | 3.0 | 2.0 | 3.0 |
| - | transcript_12987 | 373.5 | 272.1 | 277.2 | 307.6 | 1.1 | 3.9 | 3.8 | 2.9 |
| - | transcript_16122 | 41.8 | 20.1 | 53.4 | 38.4 | 0.7 | 0.9 | 0.8 | 0.8 |
| - | transcript_17920 | 389.5 | 145.2 | 257.0 | 263.9 | 1.3 | 26.2 | 3.2 | 10.2 |
| - | transcript_15896 | 641.4 | 787.2 | 833.8 | 754.1 | 1645.6 | 3122.4 | 2533.8 | 2433.9 |
| - | transcript_12688 | 25.3 | 37.1 | 30.0 | 30.8 | 63.2 | 128.6 | 96.5 | 96.1 |
| - | transcript_15627 | 358.8 | 452.1 | 340.3 | 383.7 | 667.6 | 728.9 | 1538.4 | 978.3 |

**Table S6. Expression levels of differential expressed lncRNAs**

| **Gene name** | **Gene_id** | **Transcripts Per Million (TPM)** | | | | | | | |
| --- | --- | --- | --- | --- | --- | --- | --- | --- | --- |
|  |  | **Linalool_LC15** | | | | **CK** | | | |
|  |  | **B3** | **B2** | **B1** | **Average** | **A3** | **A2** | **A1** | **Average** |
| lncRNA1 | transcript_10177 | 1.2 | 6.7 | 24.0 | 10.7 | 0.2 | 0.2 | 0.1 | 0.1 |
| lncRNA2 | transcript_1047 | 1.8 | 14.3 | 48.0 | 21.4 | 0.0 | 0.0 | 0.0 | 0.0 |
| lncRNA3 | transcript_10778 | 0.4 | 5.9 | 21.4 | 9.3 | 1.4 | 0.3 | 0.2 | 0.6 |
| lncRNA4 | transcript_10842 | 0.5 | 0.5 | 1.3 | 0.8 | 0.0 | 0.0 | 0.0 | 0.0 |
| lncRNA5 | transcript_11417 | 4.0 | 4.3 | 2.6 | 3.6 | 1.9 | 1.4 | 1.6 | 1.6 |
| lncRNA6 | transcript_11886 | 0.0 | 1.9 | 6.6 | 2.8 | 0.0 | 0.0 | 0.0 | 0.0 |
| lncRNA7 | transcript_1219 | 0.7 | 3.4 | 12.8 | 5.6 | 0.0 | 0.1 | 0.3 | 0.1 |
| lncRNA8 | transcript_1222 | 2.5 | 10.1 | 47.5 | 20.0 | 0.0 | 0.0 | 0.3 | 0.1 |
| lncRNA9 | transcript_12423 | 0.3 | 1.5 | 2.6 | 1.5 | 0.1 | 0.1 | 0.3 | 0.2 |
| lncRNA10 | transcript_12735 | 0.5 | 2.0 | 7.2 | 3.2 | 0.2 | 0.3 | 0.1 | 0.2 |
| lncRNA11 | transcript_13633 | 2.9 | 20.8 | 69.0 | 30.9 | 0.2 | 0.0 | 0.1 | 0.1 |
| lncRNA12 | transcript_1378 | 2.2 | 5.1 | 3.1 | 3.4 | 10.4 | 22.0 | 7.7 | 13.4 |
| lncRNA13 | transcript_1400 | 2.0 | 10.2 | 28.4 | 13.6 | 0.0 | 0.1 | 0.1 | 0.1 |
| lncRNA14 | transcript_14149 | 0.4 | 3.8 | 13.0 | 5.7 | 0.0 | 0.0 | 0.1 | 0.0 |
| lncRNA15 | transcript_14213 | 0.6 | 2.5 | 9.3 | 4.1 | 0.3 | 0.2 | 0.0 | 0.2 |
| lncRNA16 | transcript_14345 | 0.5 | 4.0 | 8.8 | 4.4 | 0.0 | 0.5 | 0.3 | 0.3 |
| lncRNA17 | transcript_14897 | 0.5 | 4.1 | 18.4 | 7.6 | 0.2 | 0.5 | 0.3 | 0.3 |
| lncRNA18 | transcript_14947 | 20.1 | 15.6 | 13.0 | 16.2 | 9.4 | 6.3 | 7.6 | 7.8 |
| lncRNA19 | transcript_15121 | 0.8 | 5.9 | 19.4 | 8.7 | 0.2 | 0.1 | 0.0 | 0.1 |
| lncRNA20 | transcript_15376 | 0.3 | 0.3 | 1.6 | 0.7 | 0.0 | 0.0 | 0.0 | 0.0 |
| lncRNA21 | transcript_15457 | 1.4 | 11.9 | 42.3 | 18.6 | 0.0 | 0.0 | 0.0 | 0.0 |
| lncRNA22 | transcript_15459 | 5.7 | 37.8 | 136.1 | 59.9 | 0.3 | 0.3 | 0.2 | 0.2 |
| lncRNA23 | transcript_15625 | 13.6 | 51.0 | 186.8 | 83.8 | 4.7 | 6.0 | 7.7 | 6.2 |
| lncRNA24 | transcript_15675 | 0.3 | 0.6 | 5.9 | 2.3 | 0.1 | 0.2 | 0.1 | 0.1 |
| lncRNA25 | transcript_15844 | 0.5 | 3.4 | 11.8 | 5.2 | 0.0 | 0.0 | 0.0 | 0.0 |
| lncRNA26 | transcript_1594 | 2.8 | 5.1 | 8.2 | 5.4 | 1.3 | 1.1 | 1.4 | 1.3 |
| lncRNA27 | transcript_16058 | 2.9 | 16.1 | 65.9 | 28.3 | 0.1 | 0.1 | 0.0 | 0.0 |
| lncRNA28 | transcript_16086 | 2.5 | 26.5 | 76.9 | 35.3 | 0.0 | 0.0 | 0.0 | 0.0 |
| lncRNA29 | transcript_16453 | 14.1 | 7.9 | 11.2 | 11.1 | 4.3 | 3.6 | 3.5 | 3.8 |
| lncRNA30 | transcript_16619 | 11.5 | 90.6 | 354.2 | 152.1 | 4.9 | 7.0 | 1.3 | 4.4 |
| lncRNA31 | transcript_16649 | 12.3 | 8.5 | 6.8 | 9.2 | 21.1 | 30.7 | 26.5 | 26.1 |
| lncRNA32 | transcript_16667 | 0.3 | 2.6 | 14.2 | 5.7 | 0.1 | 0.1 | 0.0 | 0.0 |
| lncRNA33 | transcript_16769 | 0.8 | 5.2 | 26.0 | 10.7 | 0.3 | 0.6 | 0.0 | 0.3 |
| lncRNA34 | transcript_16991 | 0.4 | 0.2 | 0.5 | 0.3 | 1.8 | 4.8 | 1.2 | 2.6 |
| lncRNA35 | transcript_17015 | 1.4 | 1.8 | 7.0 | 3.4 | 0.0 | 0.0 | 0.0 | 0.0 |
| lncRNA36 | transcript_17093 | 12.3 | 21.1 | 15.2 | 16.2 | 40.9 | 53.6 | 50.9 | 48.5 |
| lncRNA37 | transcript_1807 | 0.3 | 1.7 | 15.5 | 5.8 | 0.0 | 0.0 | 0.0 | 0.0 |
| lncRNA38 | transcript_18159 | 0.4 | 1.2 | 6.9 | 2.8 | 0.0 | 0.0 | 0.0 | 0.0 |
| lncRNA39 | transcript_18337 | 0.3 | 2.3 | 12.0 | 4.9 | 0.0 | 0.0 | 0.0 | 0.0 |
| lncRNA40 | transcript_18385 | 0.2 | 2.6 | 15.5 | 6.1 | 0.2 | 0.1 | 0.1 | 0.1 |
| lncRNA41 | transcript_18644 | 0.6 | 2.8 | 12.6 | 5.3 | 0.6 | 0.3 | 0.0 | 0.3 |
| lncRNA42 | transcript_18738 | 0.2 | 0.8 | 1.9 | 1.0 | 0.0 | 0.0 | 0.0 | 0.0 |
| lncRNA43 | transcript_18797 | 0.3 | 4.1 | 10.5 | 5.0 | 0.4 | 0.1 | 0.0 | 0.2 |
| lncRNA44 | transcript_19011 | 8.7 | 19.1 | 71.1 | 33.0 | 1.8 | 1.1 | 1.7 | 1.5 |
| lncRNA45 | transcript_19064 | 4.9 | 35.0 | 106.8 | 48.9 | 0.5 | 2.8 | 1.0 | 1.4 |
| lncRNA46 | transcript_19099 | 11.4 | 12.9 | 11.0 | 11.8 | 3.1 | 5.7 | 3.7 | 4.1 |
| lncRNA47 | transcript_19139 | 1.7 | 8.9 | 37.1 | 15.9 | 0.0 | 0.1 | 0.0 | 0.0 |
| lncRNA48 | transcript_19141 | 1.5 | 1.9 | 3.3 | 2.2 | 0.4 | 0.0 | 0.1 | 0.2 |
| lncRNA49 | transcript_19362 | 1.1 | 9.4 | 13.4 | 7.9 | 0.2 | 0.1 | 0.1 | 0.1 |
| lncRNA50 | transcript_19699 | 3.2 | 5.4 | 20.9 | 9.8 | 0.8 | 0.0 | 1.0 | 0.6 |
| lncRNA51 | transcript_19769 | 0.4 | 8.0 | 14.8 | 7.7 | 0.3 | 0.0 | 0.8 | 0.4 |
| lncRNA52 | transcript_19954 | 5.2 | 25.0 | 99.2 | 43.1 | 2.5 | 0.4 | 3.4 | 2.1 |
| lncRNA53 | transcript_1997 | 1.3 | 3.8 | 16.2 | 7.1 | 0.4 | 0.0 | 0.2 | 0.2 |
| lncRNA54 | transcript_20067 | 1.7 | 1.5 | 1.7 | 1.6 | 7.7 | 9.3 | 4.9 | 7.3 |
| lncRNA55 | transcript_20079 | 25.5 | 183.6 | 726.0 | 311.7 | 6.6 | 7.3 | 2.1 | 5.4 |
| lncRNA56 | transcript_20694 | 10.5 | 91.9 | 345.9 | 149.4 | 7.7 | 4.7 | 1.5 | 4.6 |
| lncRNA57 | transcript_20721 | 6.3 | 10.9 | 7.9 | 8.3 | 30.9 | 20.3 | 20.4 | 23.9 |
| lncRNA58 | transcript_20758 | 0.4 | 6.6 | 20.8 | 9.3 | 0.0 | 0.0 | 0.0 | 0.0 |
| lncRNA59 | transcript_20847 | 18.1 | 98.9 | 427.7 | 181.6 | 0.3 | 3.3 | 0.3 | 1.3 |
| lncRNA60 | transcript_2086 | 69.5 | 224.5 | 648.3 | 314.1 | 55.8 | 32.7 | 86.7 | 58.4 |
| lncRNA61 | transcript_21022 | 11.0 | 42.7 | 114.4 | 56.0 | 1.7 | 0.0 | 2.6 | 1.4 |
| lncRNA62 | transcript_21136 | 2.7 | 22.1 | 62.5 | 29.1 | 0.8 | 1.1 | 0.2 | 0.7 |
| lncRNA63 | transcript_21221 | 0.4 | 6.0 | 15.3 | 7.2 | 0.0 | 0.0 | 0.0 | 0.0 |
| lncRNA64 | transcript_21323 | 0.2 | 0.0 | 0.2 | 0.1 | 0.0 | 22.5 | 3.2 | 8.6 |
| lncRNA65 | transcript_21344 | 2.1 | 5.4 | 9.6 | 5.7 | 0.9 | 1.2 | 1.0 | 1.0 |
| lncRNA66 | transcript_21456 | 4.9 | 3.2 | 0.3 | 2.8 | 19.7 | 25.7 | 18.3 | 21.2 |
| lncRNA67 | transcript_21515 | 3.1 | 10.6 | 29.0 | 14.2 | 0.0 | 0.0 | 0.0 | 0.0 |
| lncRNA68 | transcript_21596 | 1.7 | 3.0 | 10.6 | 5.1 | 0.6 | 0.4 | 0.2 | 0.4 |
| lncRNA69 | transcript_21653 | 3.1 | 6.8 | 13.3 | 7.7 | 1.4 | 2.0 | 0.2 | 1.2 |
| lncRNA70 | transcript_21831 | 0.2 | 2.7 | 10.1 | 4.3 | 0.0 | 0.0 | 0.0 | 0.0 |
| lncRNA71 | transcript_22035 | 1.6 | 8.1 | 36.6 | 15.4 | 0.0 | 0.2 | 0.0 | 0.1 |
| lncRNA72 | transcript_2252 | 12.5 | 99.3 | 401.9 | 171.3 | 11.7 | 6.5 | 2.0 | 6.7 |
| lncRNA73 | transcript_22900 | 2.2 | 15.0 | 56.8 | 24.7 | 0.0 | 0.0 | 0.0 | 0.0 |
| lncRNA74 | transcript_22985 | 1.1 | 4.5 | 9.2 | 5.0 | 0.0 | 0.0 | 0.0 | 0.0 |
| lncRNA75 | transcript_2335 | 200.0 | 385.3 | 175.9 | 253.7 | 520.1 | 856.0 | 646.1 | 674.1 |
| lncRNA76 | transcript_2380 | 65.3 | 258.8 | 820.8 | 381.6 | 63.3 | 2.5 | 37.8 | 34.5 |
| lncRNA77 | transcript_2404 | 0.0 | 0.6 | 0.5 | 0.4 | 5.3 | 4.3 | 4.4 | 4.7 |
| lncRNA78 | transcript_2417 | 32.4 | 133.6 | 471.1 | 212.3 | 29.3 | 20.9 | 29.1 | 26.5 |
| lncRNA79 | transcript_2430 | 61.4 | 299.0 | 1101.1 | 487.1 | 24.7 | 2.5 | 26.9 | 18.0 |
| lncRNA80 | transcript_24325 | 1.0 | 8.4 | 29.3 | 12.9 | 0.0 | 0.0 | 0.0 | 0.0 |

**Table S7. Expression levels of transcription factors**

| **Gene name** | **Gene_id** | **Transcripts Per Million (TPM)** | | | | | | | |
| --- | --- | --- | --- | --- | --- | --- | --- | --- | --- |
|  |  | **Linalool_LC15** | | | | **CK** | | | |
|  |  | **B3** | **B2** | **B1** | **Average** | **A3** | **A2** | **A1** | **Average** |
| TF1 | transcript_10180 | 2.0 | 1.6 | 1.9 | 1.8 | 1.7 | 2.3 | 1.6 | 1.8 |
| TF2 | transcript_10238 | 0.5 | 0.4 | 0.5 | 0.4 | 0.4 | 0.5 | 0.2 | 0.4 |
| TF3 | transcript_1076 | 64.2 | 90.0 | 94.9 | 83.0 | 96.7 | 102.4 | 78.7 | 92.6 |
| TF4 | transcript_10774 | 0.9 | 1.5 | 1.6 | 1.4 | 1.6 | 1.6 | 1.1 | 1.4 |
| TF5 | transcript_10829 | 1.6 | 3.0 | 2.5 | 2.3 | 1.9 | 2.5 | 2.0 | 2.1 |
| TF6 | transcript_11020 | 5.0 | 8.9 | 10.1 | 8.0 | 5.6 | 6.8 | 4.4 | 5.6 |
| TF7 | transcript_11407 | 2.1 | 1.9 | 2.3 | 2.1 | 2.3 | 2.1 | 1.5 | 2.0 |
| TF8 | transcript_11688 | 51.8 | 54.9 | 62.8 | 56.5 | 61.4 | 67.7 | 54.8 | 61.3 |
| TF9 | transcript_11691 | 0.8 | 1.0 | 1.0 | 0.9 | 0.8 | 1.2 | 1.6 | 1.2 |
| TF10 | transcript_11809 | 0.3 | 0.5 | 1.7 | 0.8 | 0.9 | 0.4 | 0.3 | 0.6 |
| TF11 | transcript_11864 | 4.7 | 7.9 | 6.3 | 6.3 | 5.3 | 6.6 | 5.2 | 5.7 |
| TF12 | transcript_11876 | 1.3 | 1.2 | 1.0 | 1.2 | 1.9 | 1.7 | 1.1 | 1.5 |
| TF13 | transcript_12338 | 9.1 | 9.6 | 11.4 | 10.0 | 9.6 | 8.0 | 8.4 | 8.7 |
| TF14 | transcript_12425 | 9.2 | 12.0 | 10.9 | 10.7 | 12.3 | 14.3 | 9.5 | 12.0 |
| TF15 | transcript_12460 | 21.2 | 27.2 | 25.3 | 24.6 | 27.0 | 34.9 | 23.6 | 28.5 |
| TF16 | transcript_12701 | 3.7 | 5.1 | 2.4 | 3.7 | 3.7 | 4.3 | 3.4 | 3.8 |
| TF17 | transcript_12713 | 0.2 | 0.3 | 0.3 | 0.3 | 0.0 | 0.5 | 0.1 | 0.2 |
| TF18 | transcript_12790 | 0.0 | 0.3 | 0.3 | 0.2 | 0.2 | 0.4 | 0.2 | 0.3 |
| TF19 | transcript_12918 | 0.9 | 1.5 | 1.2 | 1.2 | 0.9 | 1.1 | 1.2 | 1.0 |
| TF20 | transcript_13280 | 14.3 | 16.7 | 17.0 | 16.0 | 19.9 | 23.0 | 15.4 | 19.4 |
| TF21 | transcript_13679 | 0.5 | 0.7 | 0.6 | 0.6 | 0.3 | 1.5 | 0.5 | 0.8 |
| TF22 | transcript_13754 | 13.6 | 19.4 | 16.7 | 16.5 | 15.2 | 16.8 | 17.3 | 16.4 |
| TF23 | transcript_13761 | 0.8 | 0.5 | 0.6 | 0.6 | 0.7 | 0.9 | 0.5 | 0.7 |
| TF24 | transcript_14238 | 0.1 | 0.1 | 0.0 | 0.0 | 0.0 | 0.1 | 0.0 | 0.0 |
| TF25 | transcript_15016 | 31.2 | 32.4 | 33.2 | 32.2 | 27.3 | 34.9 | 35.7 | 32.6 |
| TF26 | transcript_15700 | 2.8 | 3.7 | 4.4 | 3.6 | 6.0 | 5.5 | 3.4 | 5.0 |
| TF27 | transcript_1636 | 103.0 | 135.8 | 156.2 | 131.7 | 112.2 | 146.6 | 137.7 | 132.2 |
| TF28 | transcript_16884 | 4.2 | 3.0 | 4.0 | 3.7 | 2.8 | 2.8 | 2.5 | 2.7 |
| TF29 | transcript_17385 | 0.4 | 0.2 | 0.3 | 0.3 | 0.2 | 0.0 | 0.3 | 0.2 |
| TF30 | transcript_17739 | 12.1 | 7.5 | 11.0 | 10.2 | 9.5 | 9.3 | 8.0 | 8.9 |
| TF31 | transcript_17812 | 7.2 | 6.0 | 8.0 | 7.1 | 5.7 | 5.4 | 5.8 | 5.6 |
| TF32 | transcript_18130 | 5.0 | 6.6 | 4.7 | 5.5 | 5.0 | 6.7 | 3.2 | 5.0 |
| TF33 | transcript_18136 | 5.1 | 5.6 | 11.1 | 7.3 | 7.4 | 9.3 | 4.0 | 6.9 |
| TF34 | transcript_18454 | 10.5 | 13.5 | 13.0 | 12.3 | 15.1 | 18.6 | 12.1 | 15.3 |
| TF35 | transcript_18665 | 9.6 | 11.8 | 11.6 | 11.0 | 10.7 | 11.4 | 13.8 | 12.0 |
| TF36 | transcript_18804 | 0.5 | 2.5 | 0.6 | 1.2 | 1.8 | 2.2 | 0.9 | 1.6 |
| TF37 | transcript_18867 | 4.3 | 6.0 | 5.0 | 5.1 | 9.5 | 6.6 | 5.3 | 7.1 |
| TF38 | transcript_19705 | 5.4 | 6.4 | 8.6 | 6.8 | 8.7 | 5.6 | 6.3 | 6.9 |
| TF39 | transcript_200 | 103.1 | 111.9 | 104.1 | 106.4 | 127.1 | 172.2 | 113.1 | 137.5 |
| TF40 | transcript_20022 | 4.6 | 3.8 | 5.7 | 4.7 | 5.2 | 6.2 | 4.3 | 5.2 |
| TF41 | transcript_20087 | 4.7 | 3.2 | 2.8 | 3.6 | 1.2 | 3.1 | 3.0 | 2.4 |
| TF42 | transcript_20802 | 1.4 | 1.0 | 1.2 | 1.2 | 1.4 | 1.6 | 1.1 | 1.4 |
| TF43 | transcript_21079 | 1.3 | 2.3 | 2.7 | 2.1 | 1.4 | 1.6 | 2.1 | 1.7 |
| TF44 | transcript_21293 | 1.9 | 1.2 | 2.4 | 1.8 | 2.5 | 5.4 | 2.8 | 3.6 |
| TF45 | transcript_21304 | 0.7 | 1.0 | 1.3 | 1.0 | 1.0 | 1.2 | 0.5 | 0.9 |
| TF46 | transcript_21703 | 12.6 | 7.6 | 9.5 | 9.9 | 16.0 | 15.0 | 7.7 | 12.9 |
| TF47 | transcript_252 | 14.2 | 20.2 | 16.5 | 17.0 | 19.0 | 27.0 | 15.7 | 20.6 |
| TF48 | transcript_25442 | 13.1 | 34.6 | 28.1 | 25.3 | 18.1 | 11.6 | 7.5 | 12.4 |
| TF49 | transcript_264 | 1.3 | 1.2 | 2.0 | 1.5 | 0.8 | 0.5 | 0.5 | 0.6 |
| TF50 | transcript_4778 | 11.3 | 15.6 | 15.4 | 14.1 | 21.9 | 18.9 | 16.9 | 19.2 |
| TF51 | transcript_4923 | 0.2 | 0.2 | 0.3 | 0.2 | 0.2 | 0.2 | 0.1 | 0.2 |
| TF52 | transcript_4974 | 2.6 | 1.8 | 1.5 | 2.0 | 2.1 | 2.6 | 2.2 | 2.3 |
| TF53 | transcript_5094 | 16.0 | 16.8 | 10.7 | 14.5 | 18.5 | 21.2 | 12.7 | 17.5 |
| TF54 | transcript_5384 | 7.2 | 5.2 | 8.3 | 6.9 | 6.5 | 6.9 | 6.7 | 6.7 |
| TF55 | transcript_5418 | 1.8 | 3.1 | 3.3 | 2.7 | 2.4 | 3.1 | 2.6 | 2.7 |
| TF56 | transcript_5451 | 2.4 | 2.7 | 2.8 | 2.6 | 3.2 | 4.1 | 2.4 | 3.2 |
| TF57 | transcript_5520 | 3.0 | 3.1 | 2.9 | 3.0 | 2.8 | 3.2 | 2.5 | 2.8 |
| TF58 | transcript_5791 | 4.7 | 6.8 | 6.1 | 5.9 | 6.5 | 6.6 | 4.7 | 6.0 |
| TF59 | transcript_5836 | 3.0 | 4.0 | 3.6 | 3.5 | 3.6 | 2.4 | 3.0 | 3.0 |
| TF60 | transcript_5928 | 0.4 | 0.5 | 0.3 | 0.4 | 0.4 | 0.1 | 0.2 | 0.2 |
| TF61 | transcript_5943 | 1.8 | 2.4 | 2.5 | 2.2 | 1.7 | 2.0 | 1.5 | 1.8 |
| TF62 | transcript_6032 | 8.3 | 9.5 | 9.9 | 9.2 | 10.7 | 11.1 | 9.4 | 10.4 |
| TF63 | transcript_6106 | 2.8 | 3.4 | 2.8 | 3.0 | 3.8 | 4.0 | 2.1 | 3.3 |
| TF64 | transcript_6124 | 6.3 | 7.1 | 7.1 | 6.8 | 7.1 | 8.7 | 7.1 | 7.6 |
| TF65 | transcript_6258 | 0.6 | 1.1 | 0.9 | 0.9 | 0.9 | 0.9 | 1.1 | 1.0 |
| TF66 | transcript_6788 | 0.9 | 0.8 | 0.8 | 0.8 | 0.8 | 0.7 | 1.0 | 0.8 |
| TF67 | transcript_69 | 4.8 | 5.5 | 5.7 | 5.4 | 6.6 | 6.1 | 4.8 | 5.8 |
| TF68 | transcript_6959 | 0.3 | 0.6 | 0.7 | 0.5 | 0.4 | 0.7 | 0.5 | 0.5 |
| TF69 | transcript_6975 | 0.8 | 1.3 | 1.9 | 1.3 | 1.2 | 1.8 | 1.1 | 1.4 |
| TF70 | transcript_7175 | 13.2 | 13.8 | 22.1 | 16.4 | 12.2 | 12.0 | 10.4 | 11.5 |
| TF71 | transcript_7277 | 0.1 | 0.5 | 0.2 | 0.3 | 0.1 | 0.4 | 0.2 | 0.2 |
| TF72 | transcript_7452 | 5.6 | 5.8 | 7.4 | 6.2 | 6.4 | 9.3 | 4.6 | 6.8 |
| TF73 | transcript_7531 | 0.0 | 0.1 | 0.1 | 0.1 | 0.0 | 0.0 | 0.1 | 0.0 |
| TF74 | transcript_7604 | 7.4 | 11.0 | 9.5 | 9.3 | 11.2 | 10.9 | 6.5 | 9.5 |
| TF75 | transcript_7608 | 3.6 | 3.7 | 3.7 | 3.6 | 4.7 | 4.6 | 3.9 | 4.4 |
| TF76 | transcript_7701 | 4.7 | 4.8 | 4.9 | 4.8 | 5.8 | 5.6 | 4.0 | 5.2 |
| TF77 | transcript_7825 | 0.0 | 0.1 | 0.1 | 0.1 | 0.1 | 0.2 | 0.0 | 0.1 |
| TF78 | transcript_7833 | 1.3 | 1.4 | 1.2 | 1.3 | 0.8 | 0.9 | 0.7 | 0.8 |
| TF79 | transcript_7968 | 10.0 | 12.6 | 12.2 | 11.6 | 10.4 | 8.8 | 8.0 | 9.1 |
| TF80 | transcript_8084 | 1.8 | 1.1 | 1.8 | 1.6 | 1.1 | 1.6 | 1.0 | 1.2 |

**Table S8. The information of oligonucleotide primers used**

| **Gene name [ID]** | **Primer name** | **Primer sequence (5’→3’)** | **Amplification efficiency (%)** | **R^2^** |
| --- | --- | --- | --- | --- |
| GAPDH [transcript_14025] | GAPDH-F | AGCCGCTGAAGGTCCTCTTA | 101.33 | 0.9989 |
|  | GAPDH-R | CTGCAGCGGCATCAAATACG |  |  |
| 18sRNA [transcript_17623] | 18sRNA-F | GGCATCGTCCTAACACCCA | 91.59 | 0.9906 |
|  | 18sRNA-R | CCGCTAGAGGCGTTTCATC |  |  |
| RPL10 [transcript_24361] | RPL10-F | CCAAAGCCACCCCACTTTGA | 101.37 | 0.9947 |
|  | RPL10-R | GCAGATTTGCTGCATAGGTCT |  |  |
| UBQ [transcript_5290] | UBQ-F | CGCGATATCCAGGAACCCAT | 104.82 | 0.9980 |
|  | UBQ-R | TTTTCAGTGCCTTGTTGCGG |  |  |
| TBP [transcript_7389] | TBP-F | TGCTTTACATGGCACGAACG | 108.89 | 0.9994 |
|  | TBP-R | TTGCTGTGGTGTATGGACGG |  |  |
| RPS3 [transcript_15264] | RPS3-F | AACGATTGGGCATCTTCAT | 98.26 | 0.9987 |
|  | RPS3-R | CACAGGATCATTTGCTGGA |  |  |
| TF102 transcript_16042] | TF102-F | GCTCCACCGAAACAGAAGGA | 101.53 | 0.9985 |
|  | TF102-R | ACGGTTGATGAAACCATACCCA |  |  |
| TF111 [transcript_3309] | TF111-F | TGTCCGACGGCGACAGTAAT | 101.77 | 0.9986 |
|  | TF111-R | TCTCTCGCATGTTGATGCAGT |  |  |
| lncRNA77 [transcript_2404] | lncRNA77-F | ATCGTGTGTGCAACGATACTA | 105.97 | 0.9992 |
|  | lncRNA77-R | TGAAAGCTCTGGCCTTGAGAA |  |  |
| lncRNA80 [transcript_24325] | lncRNA80-F | AGCTTGCTCTAAGAAAAACGGA | 109.75 | 0.9992 |
|  | lncRNA80-R | ATATCGTGTGCGGAAGAATCG |  |  |
| CYP1 [transcript_10275] | CYP1-F | AGCCCGAATCCTAAGCAACT | 112.88 | 0.9979 |
|  | CYP1-R | ACTGCCCGGACGTAGATTTC |  |  |
| CYP3 [transcript_17583] | CYP3-F | CACTCCGACTTTACCCTCCG | 107.59 | 0.9974 |
|  | CYP3-R | ACAGTTCTGGATCCCTGTGC |  |  |
| CYP4 [transcript_22624] | CYP4-F | ATCCCAACCAATTACCGCCC | 95.28 | 0.9987 |
|  | CYP4-R | CGAAATTGTTCCCACTGCCA |  |  |
| CYP6 [transcript_4508] | CYP6-F | ACATGGATTCCCTTTGGCGA | 115.11 | 0.9968 |
|  | CYP6-R | TGCTTCATACGGAGGCTTCA |  |  |
| CYP9 [transcript_9023] | CYP9-F | ACTAGGCTCAGCAGTCTGGA | 111.43 | 0.9989 |
|  | CYP9-R | AATCTCTCGGGGTCAAAGCG |  |  |
| CYP11 [transcript_9975] | CYP11-F | TGAGACACAAGATGACGCCC | 95.28 | 0.9979 |
|  | CYP11-R | CATCCAGACGTTCGGTCGAT |  |  |
| CP12 [transcript_1427] | CP12-F | CTACGTCGCCGATGAAAACG | 100.94 | 0.9962 |
|  | CP12-R | TTCGGGTTGTTGAGGATGGG |  |  |
| CP23 [transcript_1627] | CP23-F | AATGGATTCCAACCCCAAGGA | 102.91 | 0.9998 |
|  | CP23-R | GCAATCCAATCCAAAGCCCT |  |  |
| CP27 [transcript_17003] | CP27-F | GTCGGACAGTACTCCCTCCA | 106.28 | 0.9986 |
|  | CP27-R | ATTGCGTTGAAACCGTGTCC |  |  |
| CP48 [transcript_2117] | CP48-F | GCATACGAAACCAGCAACGG | 105.05 | 0.9999 |
|  | CP48-R | GGGCCGGTGTATTGGAATTG |  |  |
| CP52 [transcript_2403] | CP52-F | AGGTCACCTAATCAACGCCG | 115.61 | 0.9995 |
|  | CP52-R | GTACACGACGCCATCAGGT |  |  |
| CP56 [transcript_413] | CP56-F | TTACCGGAGACAGCAAGAGC | 90.55 | 0.9957 |
|  | CP56-R | CGTTGAATCCGTGGATTGG |  |  |


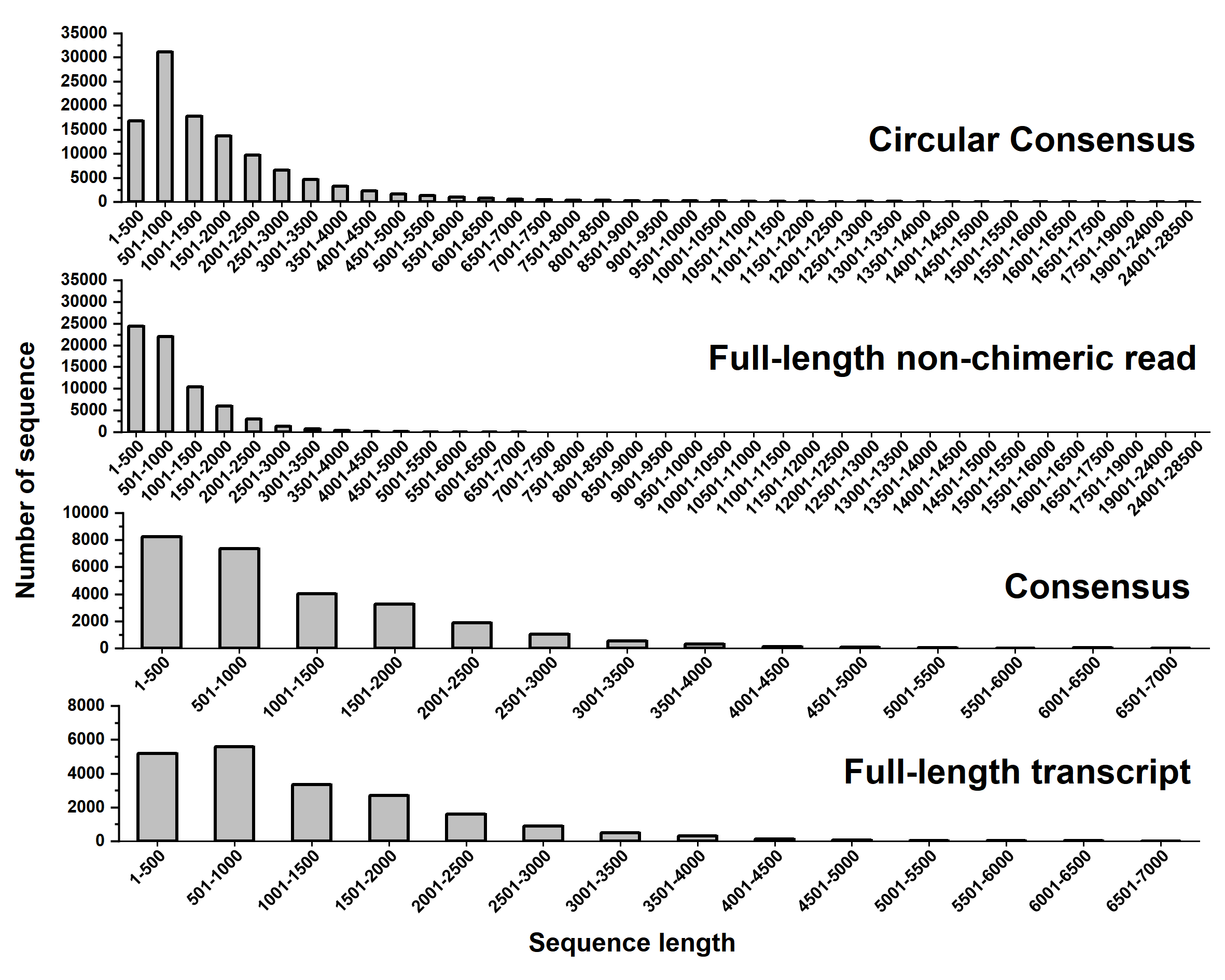


**Figure S1. Read length distribution of circular consensus, full-length non-chimeric, consensus, and full-length transcript after the filtering process**

**
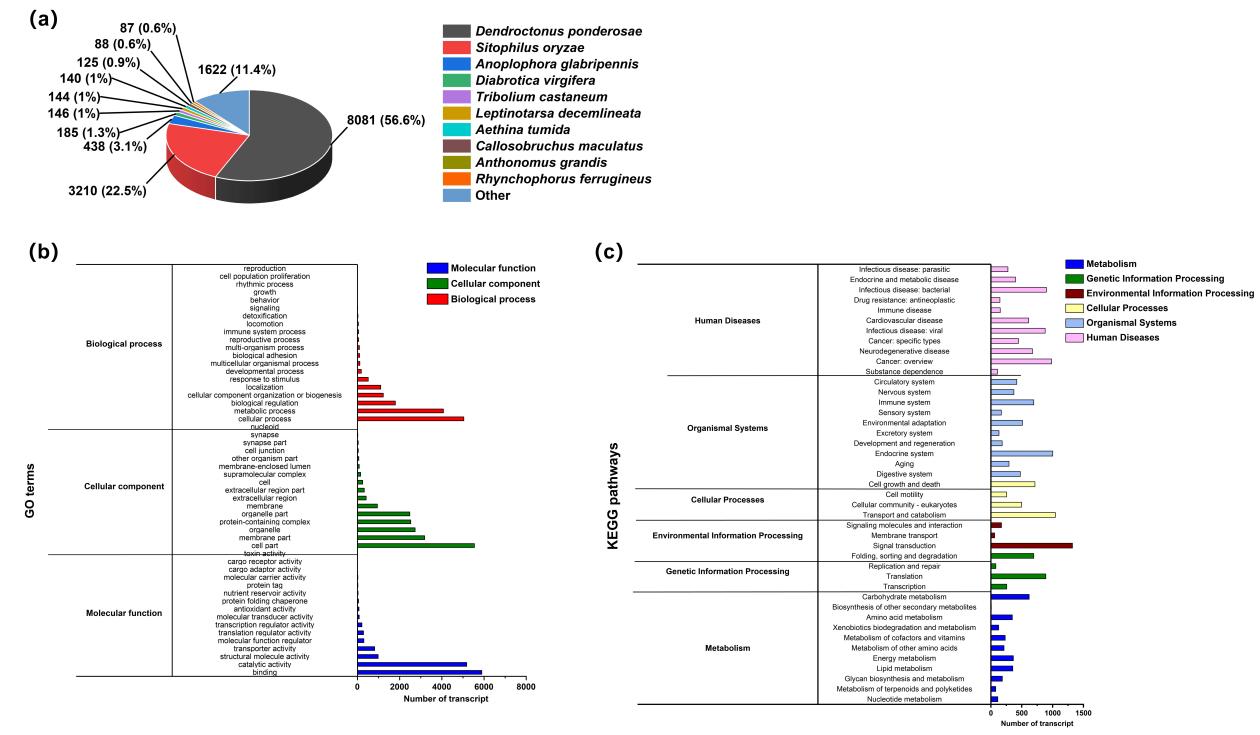
**

**Figure S2. Annotation analysis of *P. tsushimanus* transcriptome. (a)** species distribution of top blastx matches to Nr database. **(b)** GO classifications of all annotated unigenes from the transcriptome. **(c)** KEGG classifications of all annotated unigenes from the transcriptome.


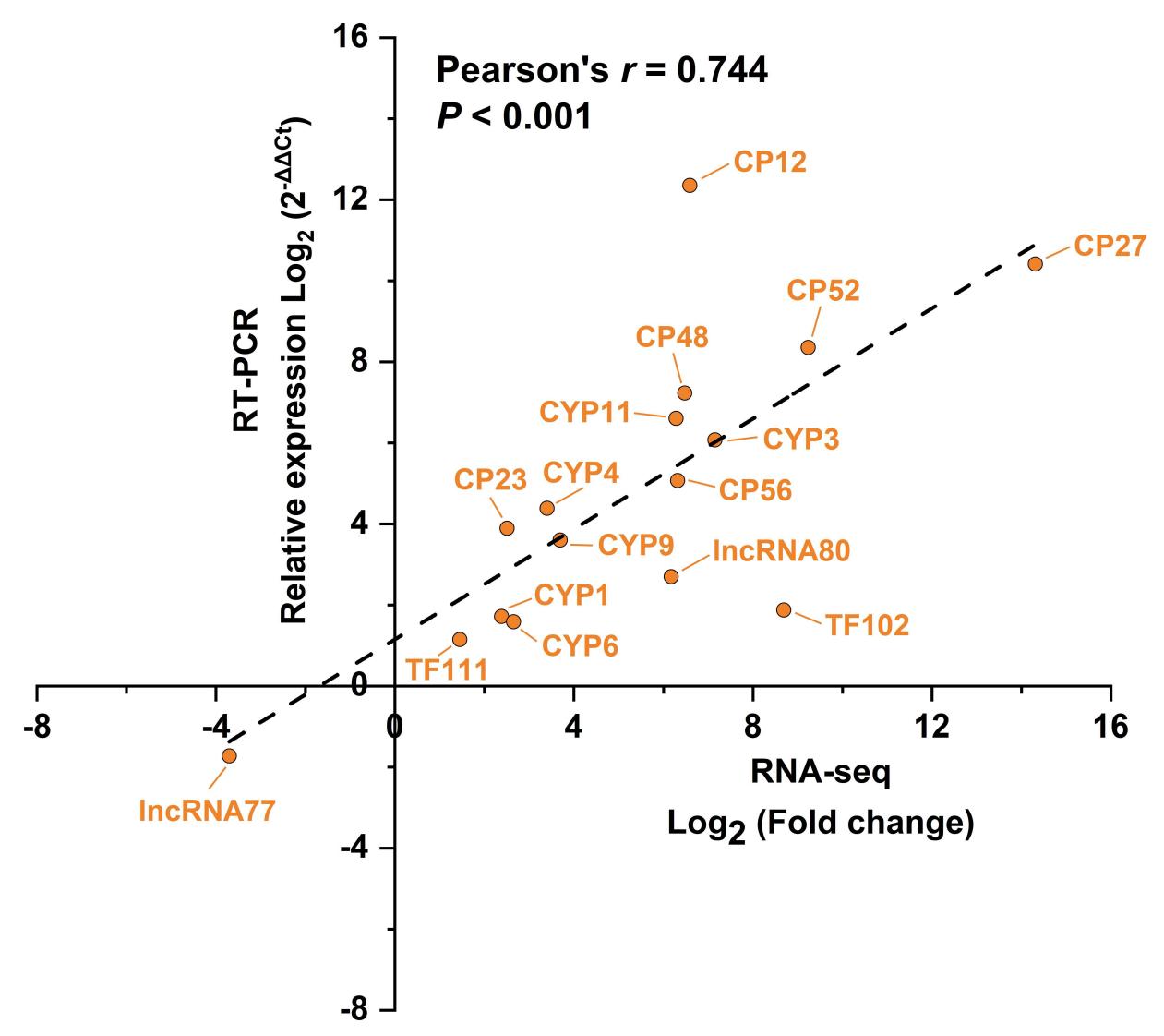


**Figure S3. Correlation analysis of RNA-Seq and RT-qPCR results using Pearson’s correlation coefficient.**
